# A New Series of Multication *M*LiZnS_2_ (M = Na, K, Rb, and Cs) Compounds for Photovoltaic Applications—A First-Principles Study

**DOI:** 10.1021/acsomega.5c06023

**Published:** 2025-10-14

**Authors:** Suresh Alagarsamy, Kanimozhi Balakrishnan, Ponniah Vajeeston

**Affiliations:** 1 Department of Physics, 566277 Maulana Azad National Institute of Technology, Bhopal, Madhya Pradesh 462003, India; 2 Department of Computational Physics, School of Physics, 29944 Madurai Kamaraj University, Palkalai Nagar,, Madurai, Tamil Nadu 625021, India; c Department of Chemistry and Center for Materials Science and Nanotechnology, 6305 University of Oslo, Oslo 0371, Norway

## Abstract

This study presents the first comprehensive first-principles investigation of the structural, dynamical, mechanical, electronic, and optical properties of *M*LiZnS_2_ (M = Na, K, Rb, and Cs), a new class of layered quaternary chalcogenides experimentally synthesized but not theoretically explored in detail. Density functional theory (DFT) calculations reveal a systematic phase transition from trigonal (NaLiZnS_2_) to tetragonal (K, Rb, and Cs analogues), driven by the ionic radius of the A-site cation. Phonon spectra confirm the dynamical stability of all equilibrium phases, while elastic constants satisfy Born criteria, verifying the mechanical stability (except tetragonal NaLiZnS_2_). Hybrid functional (HSE06) bandgaps (2.9–3.6 eV) align with related experimental trends, highlighting their suitability for optoelectronic applications. Optical analysis indicates low reflectivity, high absorption in the UV–visible range, and refractive indices compatible with antireflection coatings in photovoltaics. Electronic structures show flat valence bands and phonon gaps linked to high hole mobility and reduced thermal conductivity, suggesting prospects for spintronics and thermoelectrics. This work establishes *M*LiZnS_2_ as a promising platform for photovoltaic and multifunctional energy materials, bridging experimental synthesis and computational insights to guide future studies.

## Graphical abstract

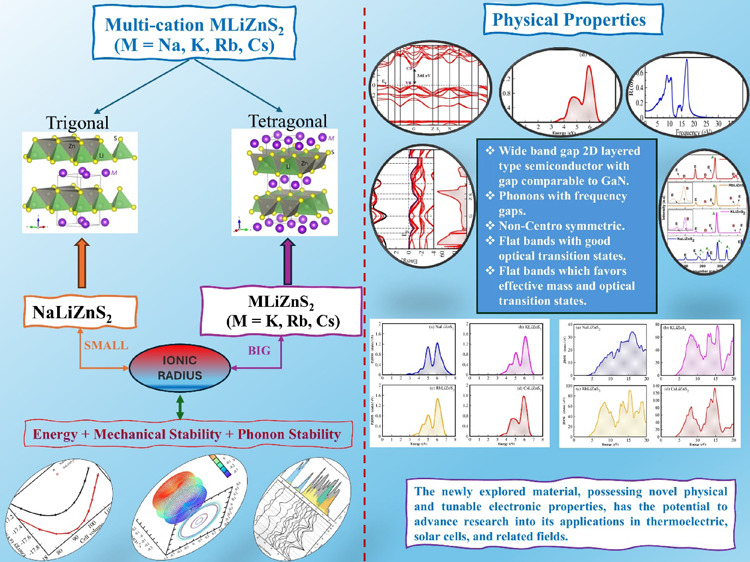

## 1 Introduction

Quaternary chalcogenides are promising materials for efficient heat-to-electricity conversion. Their ability to exhibit fast ionic diffusion, especially in trigonal phases, makes them potential solid-state electrolytes for energy storage devices like batteries. Moreover, their tunable bandgaps and optical properties make them useful in photovoltaic applications and optoelectronic devices.^1–3^ The demand for innovative technologies to replace conventional semiconductor-based systems has steadily increasing. The limitations associated with existing technologies often stem from the restricted set of materials suitable for large-scale manufacturing. Consequently, the development of next-generation technologies requires not only the synthesis of novel materials but also a comprehensive understanding of their physical properties. Recent progress in material synthesis techniques has opened new possibilities for creating materials with diverse anionic compositions, allowing the fine-tuning of their physical properties to surpass those of traditional semiconductors.^4^ For instance, Rao et al. demonstrated that the incorporation of aliovalent anions as substitutes for a single anion in ZnO significantly enhanced its physical properties compared to its parent compound.^5^ Extending upon this basis, this study explores a new series of *M*LiZnS_2_ (M = K, Rb, and Cs) to uncover its unique physical and chemical properties, paving the way for its potential use as a superior alternative to conventional materials.

The ThCr_2_Si_2_-type structure represents one of the most widely studied and fascinating layered materials that crystallizes in the tetragonal crystal system. This structural type is important because it can accommodate many different elements, leading to the creation of materials with unique properties. These properties have the potential to drive advancements in energy technologies and other emerging fields.^6^ New layered multication semiconductors CsLiCoS_2_, CsAgCoS_2_, and CsLiFeS_2_ have been synthesized using the conventional solid-state reaction method, exhibiting a small thermal activation energy gap.^7^ It is suggested that the ionic radii of the constituent elements play a key role in determining their crystal structures. Specifically, CsLiCoS_2_ adopts a disordered tetragonal structure, whereas CsAgCoS_2_ and CsLiFeS_2_ favor an ordered orthorhombic structure, resulting in distinct physical properties. In comparison, CsCuFeS_2_, which also crystallizes in a tetragonal structure, demonstrates metallic behavior as confirmed by extended Hückel tight-binding calculations. This metallic nature arises due to the significant contribution of Fe 3*d* orbitals at the Fermi level.^8^ Experimental studies reveal that ACuFeS_2_ (A = K, Rb, and Cs) exhibits semiconducting behavior, with lattice parameters increasing as the size of the alkali metal increases from K to Cs.^9,10^ Interestingly, when A is replaced by smaller alkali metals such as Li and Na, the material adopts a trigonal structure.^11^ Similarly, ACuMnSe_2_ (A = K, Rb, and Cs) also demonstrates semiconducting properties, with an increase in lattice parameters corresponding to the larger size of A.^12^ ACoCuS_2_ (A = K, Rb, and Cs), synthesized via the sulfurization method, crystallizes in a tetragonal structure and shows semiconducting transport properties.^13^ Notably, replacing A with Li or Na in such systems leads to the formation of a trigonal CaAl_2_Si_2_-type structure. Additionally, NaCuMS_2_ (M = Mn, Fe, Co, Zn) prepared using the sulfurization method crystallizes in the trigonal CaAl_2_Si_2_-type structure and exhibits semiconducting electrical properties.^14^ The electrical resistivity of ALiFeS_2_ (A = Na, K, Rb) semiconductors increases with the size of the alkali metal atom, indicating that the ionic radius plays a crucial role in determining the stability of the crystal structure and its physical properties.^15^ Additionally, three new compounds, CsLiCrSe_2_, RbLiCrS_2_, and CsLiCrS_2_ have been successfully synthesized using the conventional solid-state method. It has been reported that the S-(Cr/Li)-S bond angle restricts the formation of such materials when smaller alkali metal atoms are involved.^16^

Advancements in material synthesis have enabled the possibility of producing chalcogenides with lighter alkali atoms in recent days. We chose M = Na, K, Rb, and Cs for this study based on their experimental synthesis in *M*LiZnS_2_ phases and their systematic variation in ionic radii, which allows investigation of symmetry-driven phase transitions from trigonal to tetragonal forms.^17,18^ To the best of our knowledge, no first-principles calculation work is available in this new series of *M*LiZnS_2_ (M = Na, K, Rb, and Cs). This new series adopts a ThCr_2_Si_2_ two-dimensional-layered-type structure. In all of the ThCr_2_Si_2_-type materials, it is still a mystery why lighter metal atoms prefer lower symmetry, and heavy metal atoms prefer higher symmetry. While metal oxides like TiO_2_ and SnO_2_ are widely utilized for optical, photocatalytic, and energy-related applications due to their stability and wide bandgaps,^19,20^ recent advancements in photocatalytic materials, including perovskites, metal–organic frameworks (MOFs), and covalent organic frameworks (COFs), have expanded the possibilities for efficient light-driven processes.^21–24^ Among these, *M*LiZnS_2_ compounds stand out due to their layered structure, tunable optical properties, and ease of thin-film fabrication. These materials offer significant advantages in the design of advanced optical displays, photocatalytic systems, and flexible electronic devices. Furthermore, their synthesis is more straightforward and cost-effective compared with both conventional metal oxides and emerging photocatalysts, making them an attractive alternative for next-generation thin-film technologies. In the present new series, which have been taken for the study prefer low symmetry structure when *M* has lower mass and high symmetry for *M*LiZnS_2_ (M = K, Rb, and Cs). For the first time, DFT calculations were performed for eight structures of *M*LiZnS_2_ (M = Na, K, Rb, and Cs) by placing each structure into trigonal and tetragonal configurations. This selection enables understanding how cation size affects structural stability, bonding, and optoelectronic behavior in layered chalcogenides.

We employed DFT due to its proven accuracy and efficiency in predicting the properties of multication quaternary systems. While more advanced methods such as ab initio quantum Monte Carlo are computationally expensive, DFT offers an optimal trade-off between precision and feasibility for extended solid-state systems. Furthermore, we briefly refer to constrained DFT (CDFT) studies, which provide complementary insights into charge-transfer processes and potential energy surfaces, thereby reinforcing the theoretical understanding of such layered compounds.

The primary goal of this work is to determine the equilibrium structure of the *M*LiZnS_2_ series as no theoretical or experimental studies beyond synthesis reports currently exist for these compounds. Subsequently, we investigate their physical characteristics using DFT and compare them to conventional semiconductors such as ZnO and GaN, given that these layered materials exhibit unique properties that may open new avenues in unconventional energy-related research. To establish structural stability, we performed energy–volume, phonon dispersion, and elastic property calculations. Additionally, this study provides a comprehensive analysis of the optical properties of the *M*LiZnS_2_ (M = Na, K, Rb, and Cs) series. This computational approach greatly minimizes the time and resources required for experimental trial-and-error, thereby streamlining the design and synthesis of novel materials with tailored properties.

## 2 Computational Details

The total lattice energies of the *M*LiZnS_2_ in tetragonal and trigonal structures were calculated by DFT, as implemented in the VASP code.^25^ The interaction between the core (Li:[He], Na:[Ne], K:[Ar], Rb:[Kr], Cs:[Xe], Zn:[Ar], and S:[Ne]) and the valence electrons were described using the projector-augmented wave (PAW)^26^ method. The Perdew, Burke, and Ernzerhof (PBE)^27^ gradient corrected functional was used for the exchange-correlation part of the potential for structural optimization. In this study, we used DFT with the generalized gradient approximation (GGA) using the Perdew–Burke–Ernzerhof (PBE) functional for structural optimization, mechanical properties, and phonon calculations. The PBE functional was chosen because it gives accurate and reliable results for important structural properties such as lattice constants, bond lengths, and bulk moduli in many materials, including oxides and chalcogenides. The large energy cutoff of 600 eV was used to guarantee basis-set completeness. The atoms were deemed to be relaxed when all atomic forces were less than 0.02 eV Å^–1^, and the geometries were assumed to get optimized when the total energy converged to ≤1 meV between two consecutive geometric optimization steps. The crystal lattice parameters for all *M*LiZnS_2_ phases in equilibrium were computed accordingly.

The frozen phonon calculation was performed on these polymorphs using the Phonopy program to obtain the phonon dispersion curve and the density of states (DOS).^28^ An atomic displacement of 0.0075 Å was used, with symmetry consideration, to obtain the force constants for the phonon calculations. The displacements in opposite directions along all axes were incorporated into the calculations to improve the overall precision. The force calculations were made using the VASP code with the supercell approach, and the resulting data were imported into the Phonopy program. The dynamical matrices were calculated from the force constants, and phonon DOS curves were computed using the Monkhorst–Pack scheme.^28^ The optical characteristics were derived from the complex dielectric function of ε­(ω) = ε_1_(ω) + iε_2_(ω). In this formulation, ε_2_(ω) was determined through interband transitions utilizing Fermi’s golden rule, while ε_1_(ω) was acquired through the application of the Kramers–Kronig relations. Subsequently, the real and imaginary components of the dielectric function were employed to calculate essential optical parameters including the absorption coefficient, refractive index, reflectivity, and optical conductivity.

## 3 Results and Discussion

### 3.1 Structural Stability

DFT calculations were performed for a new quaternary sulfide series, *M*LiZnS_2_ (M = Na, K, Rb, and Cs). To determine the structural stability of these compounds, each *M*LiZnS_2_ was analyzed within two possible crystal systems: trigonal and tetragonal.

*M*LiZnS_2_ (M = Na, K, Rb, and Cs) in the trigonal structure (Figure[Fig fig1]b) with space group *P3̅m1* and one formula unit is formed by the *M* atom occupying the 1*a* Wyckoff position, Li/Zn by the 2*d* Wyckoff position with half occupancy, and the S atom by the 2*d* Wyckoff position. Li and Zn atoms are distorted equally at the 2*d* position. So, it is necessary to place the Li atom at the 1*b* Wyckoff position and Zn at the 1*c* Wyckoff position of the *P3m1* space group for the electronic structure calculation. The structure is formed in a two-dimensional layer form with an *M* atom sandwiched between two layers. The *M* atom at the 1*a* Wyckoff position is connected with six S atoms, three from the top layer, and three from the bottom layer, forming octahedra. Li and Zn atoms coordinated with four S atoms, forming tetrahedra in the structure.

**Figure 1:**
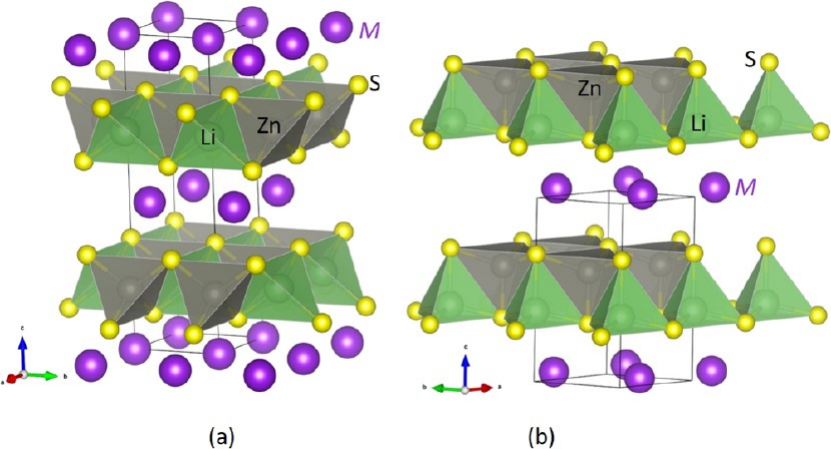
Crystal structure of *M*LiZnS_2_ (M = Na, K, Rb, and Cs) compounds in (a) tetragonal (*I4̅m2)* and (b) trigonal (*P3m1)* structures. The unit cell indicates the tetragonal structure where the *M*LiZnS_2_ series crystallizes with two formula units, whereas the trigonal structure with one formula unit.

The tetragonal structure of *M*LiZnS_2_ (M = Na, K, Rb, and Cs), shown in Figure 1a, belongs to the *I4̅m2* space group and contains two formula units (f.u.) per unit cell. This structure is analogous to the well-known layered compound ThCr_2_Si_2_. In this arrangement, the M atom occupies the 2a Wyckoff position, the Li and Zn atoms occupy the 2c and 2d Wyckoff positions, respectively, and the S atom resides at the 4e Wyckoff position. Within this structure, the *M* atom is coordinated with eight S atoms, four from the top sulfur layer, which also contains Li and Zn atoms, and four from the bottom sulfur layer is forming a bipyramidal coordination environment. Meanwhile, both Li and Zn atoms form tetrahedral units by bonding with four S atoms.

The calculated *E*–*V* curves for the new *M*LiZnS_2_ series are shown in Figure 2. Specifically, for NaLiZnS_2_ (Figure 2a), the energy minimum of the trigonal crystal structure is found to be lower than that of the tetragonal structure. This structural preference is primarily due to the influence of the ionic size and electrostatic interactions. Na, being a lighter and smaller alkali metal compared to other possible M-site elements, stabilizes the trigonal coordination by optimizing the lattice strain and minimizing the overall structural distortion. Consequently, the trigonal phase is energetically more favorable for NaLiZnS_2_ than the tetragonal configuration. This result aligns well with previous findings reported by Deng et al.^17^ Conversely, for *M*LiZnS_2_ compounds containing heavier alkali metal atoms (M = K, Rb, and Cs) (Figure 2b–d), the tetragonal structure exhibits the lowest energy, signifying a strong preference for the tetragonal system. As the alkali metal atom’s size increases, the structural stability shifts from the trigonal to the tetragonal phase, accompanied by an increase in the volume of the most stable structure. For NaLiZnS_2_, the energy difference between the trigonal and tetragonal structures is relatively large, at 0.38 eV. However, as the alkali metal size increases, this difference decreases significantly. In the case of KLiZnS_2_, the energy gap is reduced to 0.1 eV, leading to a sudden shift in the minimum energy structure, favoring the tetragonal phase. In RbLiZnS_2_, the energy difference between the trigonal and tetragonal structures increases slightly compared to that in KLiZnS_2_, and this difference reaches its maximum in CsLiZnS_2_, further reinforcing the preference for the tetragonal phase. The *E*–*V* curves further confirm that NaLiZnS_2_ preferentially crystallizes in the trigonal structure, whereas *M*LiZnS_2_ compounds with heavier alkali metals (M = K, Rb, and Cs) adopt the tetragonal structure. These findings align exceptionally well with experimental observations.^6,8,11,17^ The *E*–*V* curve can also be used to predict the high-pressure and metastable structures of the materials. Furthermore, the energy vs volume curves for these structures do not intersect at any point, indicating that no metastable structure is possible under the investigated conditions. In addition, recent first-principles studies reported by Sergio Conejeros et al. confirm that ACuFeS_2_ phases exhibit a strong dependence on A-site cation size, where smaller cations like Li favor the trigonal CaAl_2_Si_2_-type structure, while larger cations like K stabilize the tetragonal ThCr_2_Si_2_-type structure. These calculations also predict fast copper ion diffusion in the trigonal phase derived from LiCuFeS_2_, which is absent in the tetragonal KCuFeS_2_ phase.^9^

**Figure 2:**
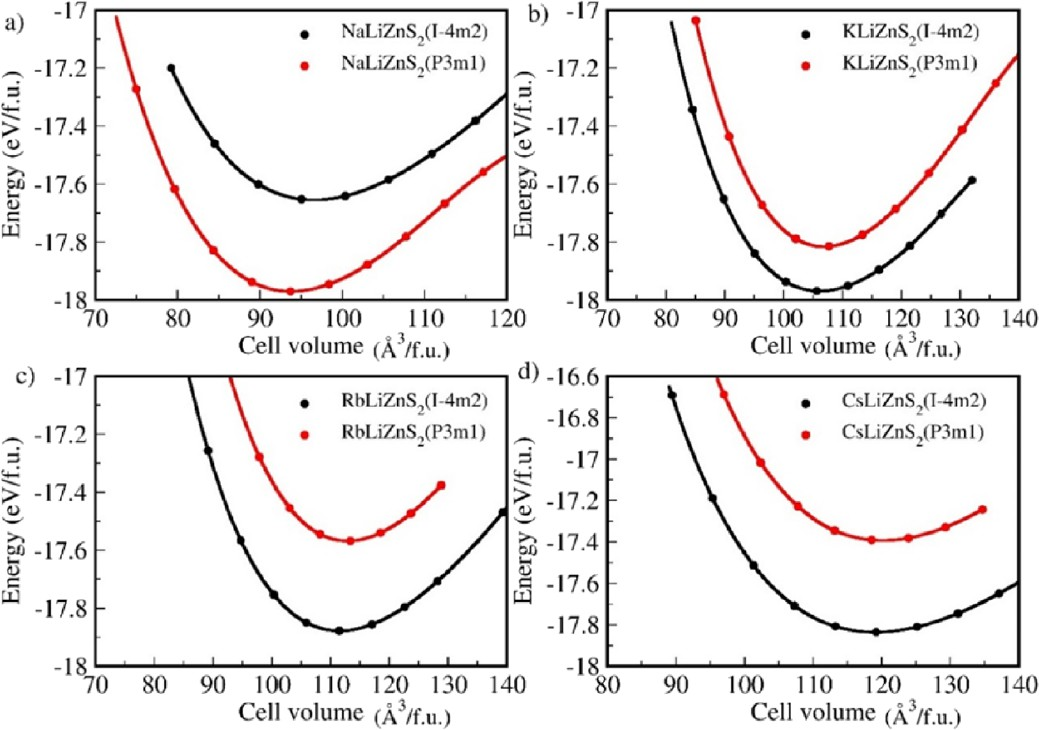
Calculated total energy as a function of unit cell volume for *M*LiZnS_2_ (M = Na, K, Rb, and Cs). (a) NaLiZnS_2_, (b) KLiZnS_2_, (c) RbLiZnS_2_, and (d) CsLiZnS_2_. Energy and volume are normalized per formula unit (f.u.). The black curve shows the tetragonal structure, and the red curve shows the trigonal structure for all four compounds.

Table 1 presents the lattice parameters derived from DFT calculations at equilibrium alongside the corresponding experimental values. The calculated lattice parameters are compared with the reported experimental results, which vary from 0.5 to 1.9% from the reported values. In the tetragonal structure of *M*LiZnS_2_ (M = K, Rb, and Cs), single M–S, Li–S, and Zn–S bonds are observed, with their lengths progressively increasing as the alkali metal size increases. Each sulfur (S) atom bonds with M, Li, and Zn but exhibits shorter bond lengths with Zn and Li compared to M. Although Zn and Li have different ionic radii and electronegativity, the lengths of the Li–S and Zn–S bonds are found to be equal. For KLiZnS_2_, the S–Li/Zn bond is approximately 1 Å shorter than the M–S bond, highlighting the stronger bonding of S with Zn and Li than with M. This difference increases with the alkali metal size, reaching 1.1 Å for RbLiZnS_2_ and 1.3 Å for CsLiZnS_2_. The tetrahedral coordination of Li and Zn with four sulfur atoms results in two distinct bond angles: S–Zn/Li–S = 108.47 and 111.50° in KLiZnS_2_. As M transitions from K → Rb → Cs, these angles adjust to accommodate structural changes, measured at 107.77 and 112.94° for RbLiZnS_2_, and further shifting to 106.87 and 114.82° for CsLiZnS_2_. The incorporation of heavier alkali atoms (Rb and Cs) leads to an overall expansion in M–S, Li–S, and Zn–S bond lengths. To maintain structural stability, the S–Zn/Li–S bond angles adjust accordingly. Additionally, the intralayer distance increases progressively with the inclusion of heavier alkali metal atoms, reflecting the structural adaptation necessary to accommodate their larger ionic radii.

**Table 1:** Lattice Parameters, Total Energy, and Energy/f.u of the Equilibrium Structure of the *M*LiZnS_2_ (M = Na, K, Rb, and Cs) Series

compound	crystal system	lattice parameters (Å)	total energy (eV)	energy/f.u. (eV)
NaLiZnS_2_ (Exp.)	trigonal	*a* = 3.971		
		*c* = 6.718 ^17^		
NaLiZnS_2_	trigonal	*a* = 3.976	–21.063	–4.213
		*c* = 6.846		
KLiZnS_2_ (Exp.)	tetragonal	*a* = 3.840 ^18^		
		*c* = 13.270		
KLiZnS_2_	tetragonal	*a* = 3.9749	–42.037	–4.203
		*c* = 13.372		
RbLiZnS_2_	tetragonal	*a* = 4.0256	–41.872	–4.187
		*c* = 13.760		
CsLiZnS_2_	tetragonal	*a* = 4.084, *c* = 14.288	–41.849	–4.184

Yuan et al. reported that in the ALiFeSe_2_ (A = Na, K, Rb) series, placing Na in the A-site causes significant bond elongation between Na–Se and increases the interlayer distance, destabilizing the tetragonal system and favoring the trigonal system.^15^ A similar trend is observed in the *M*LiZnS_2_ (M = Na, K, Rb, and Cs) series, which are layered materials. These compounds tend to adopt either ThCr_2_Si_2_-type (tetragonal) or CaAl_2_Si_2_-type (trigonal) structures, depending on the size of the alkali atom (M). When M is a smaller alkali atom (Na), the trigonal structure is preferred, while replacing M with heavier alkali atoms (K, Rb, and Cs) stabilizes the tetragonal structure. This behavior underscores the critical role of the ionic radius in determining the crystal structure and stability of these 2D-layered systems.

Constrained Density Functional Theory (CDFT) offers a compelling extension to standard DFT, particularly for modeling quaternary chalcogenide materials (e.g., Cu_2_ZnSnS_4_ and Cu_2_ZnGeS_4_) where localized charge or spin configurations such as defect states, magnetic centers, or carrier-transfer events play a central role.^29^ Most DFT calculations focused on the ground-state energetics and related properties. In materials that contain multication, CDFT could improve the description of polaron localization, transition-metal defect states, or exchange coupling between multiple magnetic centers scenarios where standard DFT often under-delocalizes or over-delocalizes electrons when it comes to materials like quaternary chalcogenides. For deeper insights into localized electronic states, defect behavior, and magnetic exchange interactions in the *M*LiZnS_2_ semiconductor, we recommend researchers to use CDFT as a complementary tool for effective for exploration of charge-transfer processes, where standard DFT often struggles to accurately localize electrons or spins.

### 3.2 Phonon Spectrum

The phonon spectra of all eight structures were computed to evaluate the system’s dynamical stability, as illustrated in Figure 3. The phonon spectrum is a graphical representation of the vibrational frequencies of phonons across different symmetry points in the Brillouin zone of a crystal. Dynamical stability of the compounds was determined by analyzing the frequencies in the phonon dispersion spectrum. Any high-symmetry point in the phonon dispersion spectrum exhibiting an imaginary frequency is a clear indication of thermodynamic instability. This implies that the structure is not in thermodynamic equilibrium and consequently cannot serve as a stable crystalline structure. In this study, we focused exclusively on the nonmagnetic (NM) configuration, as the *M*LiZnS_2_ compounds do not contain partially filled d- or f-orbitals that typically lead to magnetic behavior. There is no experimental or theoretical indication of magnetic ordering in these materials. Accordingly, the ferromagnetic and antiferromagnetic phases were not considered. The phonon dispersion for the NM phase shows no imaginary frequencies, confirming the dynamical stability of the system in its nonmagnetic ground state.

**Figure 3:**
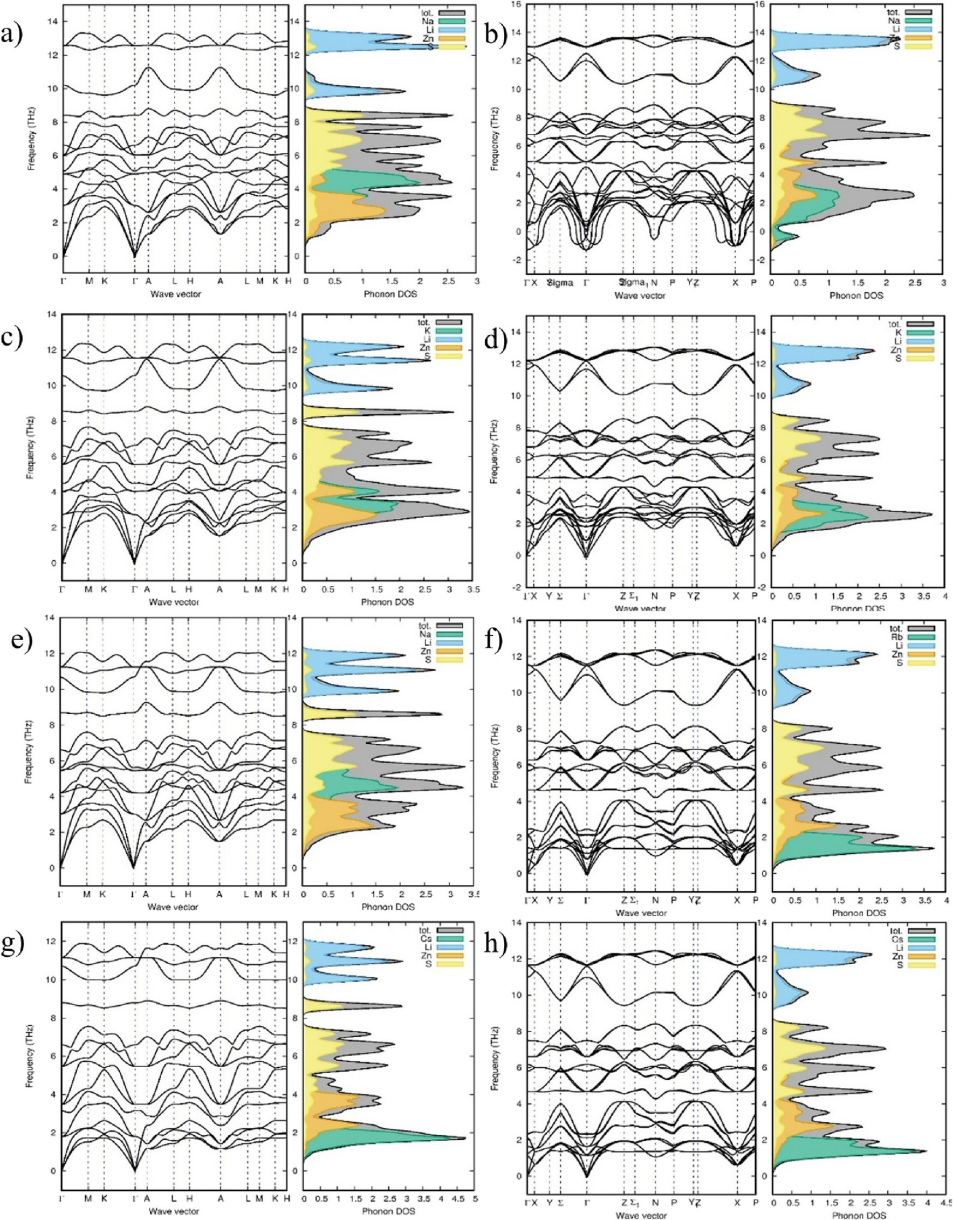
Phonon spectrum for all eight structures for the *M*LiZnS_2_ series. From top to bottom, all left side images are phonon spectra for trigonal structures of (a) NaLiZnS_2_, (c) KLiZnS_2_, (e) RbLiZnS_2_, and (g) CsLiZnS_2_. From top to bottom, all right-side images are phonon spectra for tetragonal structures of (b) NaLiZnS_2_, (d) KLiZnS_2_, (f) RbLiZnS_2_, and (h) CsLiZnS_2_.

The positive frequencies in the trigonal phonon spectrum confirm that the NaLiZnS_2_ structure is most stable in its trigonal form (Figure 3a,b shows the phonon spectra for both the trigonal and tetragonal structures of NaLiZnS_2_). The trigonal structure has 15 vibrational modes at the Γ point due to the single formula unit in the unit cell: 12 of these are optical modes, and three are acoustic. The three acoustic modes, observed in the frequency range of 0–3 THz, are primarily attributed to the vibrations of Zn atoms. These low-frequency vibrations are dominated by heavier atoms like Zn, as their greater inertia results in a slower response to perturbations. Due to their relatively low mass compared to Zn, the vibrations of Na atoms appear in the lower optical frequency range from 3 to 6 THz. The S atoms contribute in the middle range, around 6–8 THz, and the Li atoms in the higher range, from 10 to 13 THz. Lighter atoms like Li exhibit higher-frequency modes from 10 to 13 THz due to their ability to oscillate more rapidly than the other two atoms.

The tetragonal structure of NaLiZnS_2_ is determined to be unstable, as indicated by the presence of imaginary frequencies at the Γ, N, and X high-symmetry points in its phonon spectrum (Figure 3b). Imaginary frequencies signify structural instability, suggesting that this configuration cannot serve as an equilibrium structure for NaLiZnS_2_. The Na atom, located in a specific high-symmetry position within the tetragonal lattice (Wyckoff 1a), predominantly contributes to the acoustic phonon modes. Due to its low atomic mass, Na atoms introduce soft modes that could result in instabilities, reflected as imaginary frequencies in the acoustic region of the phonon spectrum. Such soft modes often occur when the restoring forces for atomic vibrations are insufficient to maintain stability. Zn and S atoms, with relatively higher masses, contribute to vibrations in the midfrequency range between 2 and 8 THz. The Li atoms, being lightweight and highly mobile due to their low mass, contribute to the high-frequency optical modes in the 10–13 THz range.

The *M*LiZnS_2_ structures (M = K, Rb, and Cs), with the M atom position occupied by heavy alkali atoms, exhibit positive frequencies in both trigonal and tetragonal configurations. This indicates that both structures are dynamically stable, although the tetragonal structure is energetically preferred for the heavy alkali atoms. In the tetragonal structure of *M*LiZnS_2_, the phonon spectrum is divided into three distinct regions: acoustic modes, midfrequency optical modes, and high-frequency optical modes. The acoustic phonons in the tetragonal structure are predominantly influenced by the *M* atom, which replaces the Zn atom’s role in the trigonal structure. The lower frequency region (0–4 THz) is primarily contributed by Zn and *M* atoms, the middle region (4–8 THz) is dominated by the S atom, and the higher frequency range (10–12 THz) arises mainly from the Li atoms. For KLiZnS_2_, phonon calculations confirm dynamic stability in both trigonal and tetragonal structures, aligning with the energy-volume curve, where the energy difference between the two structures is approximately 100 meV. The tetragonal structure exhibits two well-separated acoustic and optical regions. In this structure, the semiconductor bandgap prevents the decay of optical phonons into acoustic phonons, allowing the energy of the optical phonons to be reabsorbed by electrons. This distinct separation between acoustic and optical modes, coupled with the mixing of low-frequency optical modes with acoustic modes, suggests that the tetragonal phase of these materials holds potential for photovoltaic applications.

In polar solids, the electric dipole moment associated with long-wavelength longitudinal optical (LO) modes generates an internal electric field, causing optical phonon modes to split into longitudinal optical (LO) and transverse optical (TO) modes at the Brillouin zone center due to long-range Coulomb interactions. This LO-TO splitting, a characteristic feature of ionic bonding, is observed in NaLiZnS_2_ at approximately 12.5, 6, 5, and 3 THz along the Γ to M direction and in the tetragonal structure of KLiZnS_2_ at 2, 4, 6, and 12 THz along the Γ to X direction. However, in CsLiZnS_2_, LO-TO splitting disappears at 2 and 4 THz, indicating a transition from ionic to covalent bonding as the mass and size of the alkali atom (M) increase. Phonon density of states (DOS) analysis reveals that the 2 and 4 THz regions, where M, S, and Zn contribute to phonons, are critical in this transition, showing that bonding between M and S becomes more covalent as M increases in size. Overall, the presence of LO-TO splitting highlights the ionic bonding nature and semiconducting properties of these materials, while its gradual reduction across the series reflects the increasing dominance of covalent interactions.

The significant mass differences among *M*, Zn, Li, and S in these compounds result in three distinct phonon regions separated by two phonon gaps. In the 2–4 THz range, strong hybridization between low-frequency optical phonons and acoustic phonons is observed, as indicated by the partial density of states (PDOS), which attributes these phonons to contributions from *M*, Zn, and S atoms, highlighting strong interactions among these elements. In thermoelectric materials, phonon frequency gaps play a critical role in reducing thermal conductivity by blocking thermal phonons from propagating through the material. This response is evident in the current series, where two phonon gaps are identified, suggesting a potential for reduced phonon thermal conductivity in these materials, an important feature for thermoelectric applications.

### 3.3 Mechanical Property

Mechanical stability determines a material’s ability to maintain structural integrity in the face of deformation and load. Mechanically unstable single crystals are prone to failure or distortion, which can be costly in the design of novel materials. The stiffness of a single crystal is measured by elastic constants, which are derived from the strength of the chemical bonds and the arrangement of atoms within the crystal. Materials possessing elevated single crystal elastic constants exhibit greater rigidity and resistance to deformation, hence enhancing their mechanical stability. Materials having low single crystal elastic constants are more susceptible to deformation and may have worse mechanical stability. In general, the elastic constants of a single crystal material can be utilized to forecast its mechanical stability and to engineer materials with precise mechanical characteristics. It is crucial to comprehend the connection between single crystal elastic constants and mechanical stability to design and optimize materials for particular applications.

For a crystal structure to be stable, it must meet three key criteria: energy efficiency, thermodynamic stability, and mechanical stability. To evaluate the mechanical stability of the eight structures, their elastic properties were calculated by using the finite displacement method. Figures 4 and 5 display the computed elastic characteristics, which are reported in Table 2.

**Table 2:** Elastic Constants, Shear Modulus (*G*), Young’s Modulus (*E*), Bulk Modulus (*B*), and Poisson’s Ratio (σ) of *M*LiZnS_2_ (M = Na, K, Rb, and Cs) Series Obtained from Voigt Approximations

elastic coefficients	NaLiZnS_2_ tetragonal	NaLiZnS_2_ trigonal	KLiZnS_2_ tetragonal	KLiZnS_2_ trigonal	RbLiZnS_2_ tetragonal	RbLiZnS_2_ trigonal	CsLiZnS_2_ tetragonal	CsLiZnS_2_ trigonal
C_11_	88.18	86.14	79.16	70.79	74.31	65.81	69.27	63.85
C_12_	7.02	31.18	7.28	24.02	8.00	22.60	8.65	22.37
C_13_	11.94	17.98	12.98	16.15	13.86	16.32	15.22	17.05
C_14_	0.00	0.24		5.19	0.00	6.13	0.00	7.03
C_22_	88.18	86.14	79.12	24.02	74.31	65.81	69.27	63.85
C_23_	11.94	17.98	12.98	70.79	13.86	16.32	15.22	17.05
C_33_	67.58	62.71	50.06	68.62	46.81	73.63	45.38	82.84
C_24_	0.00	–0.24		–5.19	0.00	–6.13	0.00	–7.03
C_44_	–12.46	18.12	6.76	21.62	11.12	21.89	14.25	22.34
C_55_	–12.46	18.12		21.62	11.12	21.89	14.25	22.34
C_66_	14.58	27.48	19.70	23.38	20.68	21.61	21.15	20.74
*E* (GPa)	502.55	58.65	38.62	56.72	44.13	54.93	46.33	54.87
*G* (Gpa)	–67.30	23.34	15.10	22.95	17.69	22.17	18.80	22.05
*B* (GPa)	33.82	40.16	30.08	35.80	29.22	35.08	28.81	35.83
σ	–1.99	0.26	0.29	0.24	0.25	0.24	0.23	0.25
*A* ^u^	–0.31	0.66	2.73	0.92	0.34	1.01	0.47	1.08
Debye temperature (K)		357.81	280.80	345.27	271.57	304.58	256.53	277.92
*B*/*G*	–0.503 brittle	1.721 brittle	1.992 ductile	1.560 brittle	1.652 brittle	1.582 brittle	1.532 brittle	1.625 brittle

**Figure 4:**
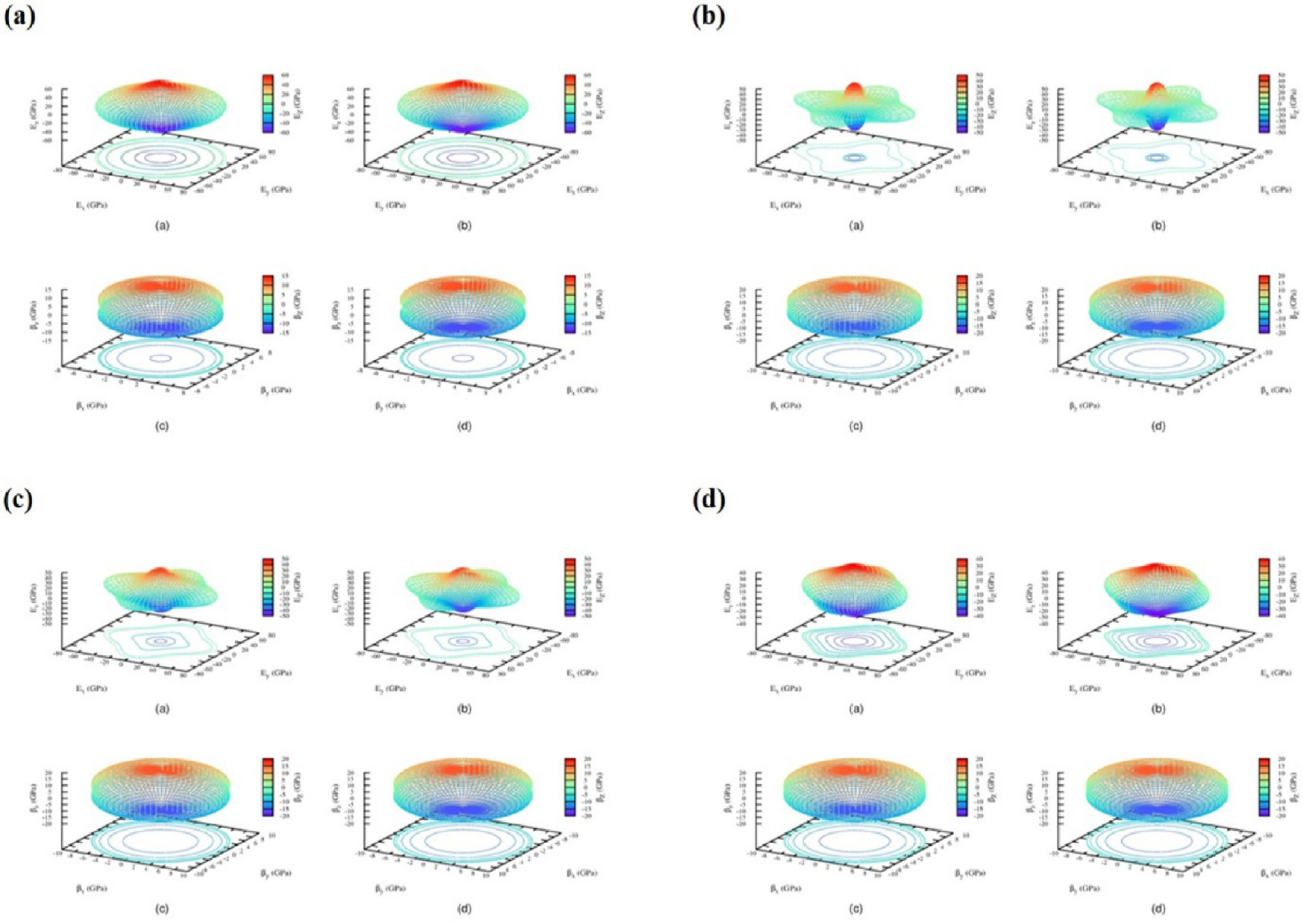
3D spatial dependence of the Young’s modulus of all four structures of for the *M*LiZnS_2_ series. Panel (a) is for the trigonal structure of NaLiZnS_2_. (b) The bottom left image is for the tetragonal structure for KLiZnS_2_. (c) The bottom left image is for the tetragonal structure for RbLiZnS_2_. (d) The bottom right image is for the tetragonal structure for CsLiZnS_2_.

**Figure 5:**
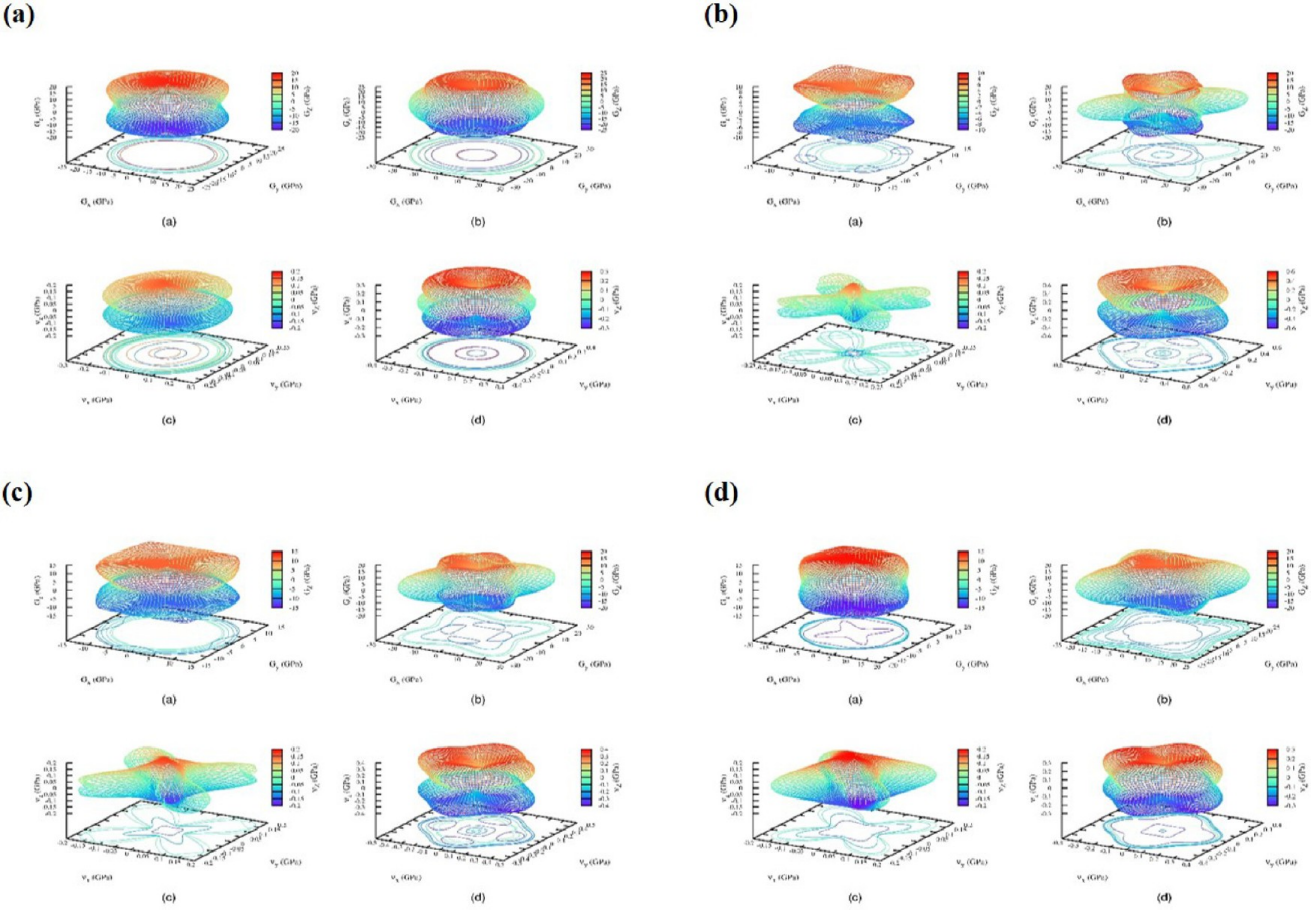
3D spatial dependence of the shear modulus of all four structures for the *M*LiZnS_2_ series. Panel (a) is for the trigonal structure of NaLiZnS_2_. (b) Top right-side image is for the tetragonal structure for KLiZnS_2_. (c) Bottom left side image is for the tetragonal structure for RbLiZnS_2_. (d) Bottom right image is for the tetragonal structure for CsLiZnS_2_.

In general, the elastic properties of a material in bulk solids are determined using Hooke’s law. This involves calculating the linear response of the stress tensor (σ) to an externally applied strain (ε) using the following formula.

$$c_{ijkl}=\frac{\partial \sigma_{ij}}{\partial \varepsilon_{ijk}}$$ 1

To be mechanically stable, it should satisfy Born stability criteria^30^

(i) C_11_–C_12_ > 0; (ii) C_13_^2^ < (1/2)*C_33_(C_11_ + C_12_); (iii) C_14_^2^ < (1/2)*C_44_*­(C_11_–C_12_) = C_44_*C_66_; (iv) C_44_ > 0.

The tetragonal structure of NaLiZnS_2_ fails to satisfy the fourth Born stability criterion, indicating that it is mechanically unstable and cannot be considered an equilibrium structure. The Born stability criteria are a set of conditions based on elastic constants that a material must fulfill to ensure its mechanical stability. When these criteria are not met, the structure is prone to mechanical failure under deformation and cannot exist as a stable phase.

In contrary, for the other *M*LiZnS_2_ compounds (where M = K, Cs, and Rb), the tetragonal structure is both energetically and thermodynamically favorable, meaning that it has a lower total energy compared to other potential structures and remains stable under thermal conditions. Moreover, these compounds satisfy all the Born stability criteria, confirming their mechanical stability. The calculated elastic constants meet the necessary conditions for stability, ensuring that the structures can withstand external forces and maintain equilibrium without undergoing deformation or collapse.

Material ductile/brittleness property is given by Pugh’s criteria,^31^ which says that it is ductile if *B*/*G* > 1.75 and brittle if *B*/*G* < 1.75. According to this condition, the KLiZnS_2_ equilibrium structure where trigonal to tetragonal transition happens in this series is ductile and all other structures are brittle in nature. Even though the NaLiZnS_2_ has a different crystal structure, it also shows brittleness. Ionic materials generally show brittleness in nature, and all materials in this new series show brittleness.

Based on the Voigt–Reuss–Hill approximate,^32,33^ mechanical parameters such as bulk modulus *B*, sheared modulus *G*, Young’s modulus *E,* and Poisson’s ratio ν are determined. From the results of the elastic constants, Young’s modulus (*E*) and Poisson’s ratio (ν) of the polycrystalline materials from Voigt–Reuss–Hill approximate^32,33^ are expressed as follows,

$$B=\frac{1}{2}(B{-}_{V}-B_{R})$$ 2

$$G=\frac{1}{2}(G{-}_{V}-G_{R})$$ 3

$$E=\frac{9BG}{3B+G}$$ 4

$$\nu =\frac{3B-2G}{2\left(3B+G\right)}$$ 5

where *B* is the bulk modulus, *G* is the shear modulus, *E* is Young’s modulus, and ν is Poisson’s ratio.

Young’s modulus is a measure of stiffness of the solids, and plastic deformation is measured by the shear modulus. NaLiZnS_2_ exhibits the highest Young’s modulus among the four materials in the series, while the values for KLiZnS_2_, RbLiZnS_2_, and CsLiZnS_2_ gradually increase from 38.620 to 46.327 GPa as the size of the M atom increases. The same trend is followed in the shear modulus and bulk modulus. Young’s modulus value for ionic solids ranges between 30 and 70 GPa. Here, the calculated value for all the structures in the series indicates ionic bonding between atoms. KLiZnS_2_ has more elasticity than the other structures as Poisson’s ratio of KLiZnS_2_ tetragonal is above 0.27. The result of Poisson’s ratio agrees well with the *B*/*G* ratio.

The anisotropy of mechanical properties is described by index *A* ^u^, if this value is higher and deviated from 0, then the structure has more anisotropy. Here, KLiZnS_2_ tetragonal is highly anisotropic (2.732) compared to the other crystals and for RbLiZnS_2_-tetragonal the value of *A* ^u^ is 0.3, which possesses the smallest anisotropy.

### 3.4 Electronic Structure

The electronic structures for the series, which are energy-wise and thermodynamically stable structures, are shown in Figure 6. The band structure is calculated for the equilibrium structure for all four materials. *M*LiZnS_2_ (M = Na, K, Rb, and Cs) show semiconducting electrical property with bandgap values of 2.91, 3.58, 3.55, and 3.61 for NaLiZnS_2_, KLiZnS_2_, RbLiZnS_2_, and CsLiZnS_2_, respectively (Table 3). In an earlier report, Deng et al.^17^ reported a bandgap value of 2.37 eV for NaLiCdS_2_, which was calculated by GGA pseudopotential, and it was finally concluded that NaLiZnS_2_ have an isostructural property as that of NaLiCdS_2_. Considering earlier reports that NaLiZnS_2_ and NaLiCdS_2_ exhibit isostructural properties, our calculated bandgap value for NaLiZnS_2_, obtained by using the hybrid pseudopotential (HSE06), accurately predicts its bandgap. Meanwhile, the other structures in this study are reported for the first time. Also, flat bands are observed in NaLiZnS_2_ at the valence band from Γ to *A*. The flat band at the valence band means low effective mass, and thus, this material has larger hole conductivity at the valence band. The KLiZnS_2_ tetragonal structure shows an indirect-type transition where the CBM occurs at Γ and the VBM occurs along Γ to *Z* path. The energy difference between the VBM and second VBM value at Γ is less than a few meV, indicating a possible direct-type transition by adding impurities. However, the other two compounds RbLiZnS_2_ and CsLiZnS_2_ show direct-type transition at the Γ point. For *M*LiZnS_2_ (M = K, Cs, and Rb), no previous report is available to compare the band structure and the bandgap values.

**Figure 6:**
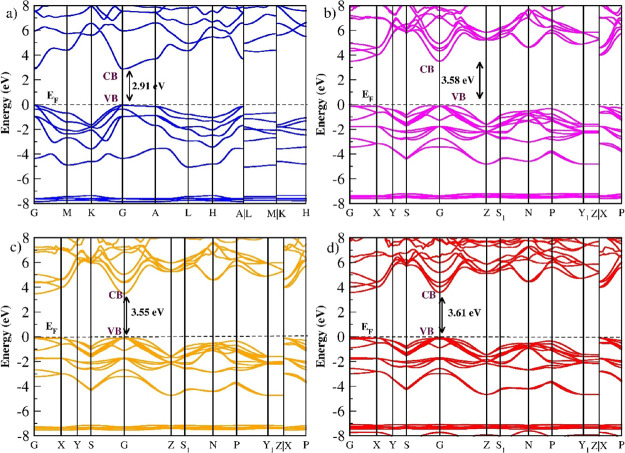
Equilibrium band structures of the *M*LiZnS_2_ series. (a) Top left image is for the trigonal structure of NaLiZnS_2_. (b) Top right-side image is for the tetragonal structure for KLiZnS_2_. (c) Bottom left side image is for the tetragonal structure for RbLiZnS_2_. (d) Bottom right-side image is for the tetragonal structure for CsLiZnS_2_.

**Table 3:** Calculated Electronic Band Structures for *M*LiZnS_2_ (M = Na, K, Rb, and Cs) Series Using HSE06 Pseudopotential Compared with Available Reports

source	method employed	bandgap (eV)	previous electronic structure availability	direct or indirect bandgap
Deng et al. (NaLiCdS_2_/NaLiZnS_2_)	GGA	2.37		direct
NaLiZnS_2_	HSE06	2.91	Deng et al.^17^	direct
KLiZnS_2_	HSE06	3.58		indirect
RbLiZnS_2_	HSE06	3.55		direct
CsLiZnS_2_	HSE06	3.61		direct

**Table 4:** Calculated Carrier Effective Mass for the *M*LiZnS_2_ (M = Na, K, Rb, and Cs) Series

	electron effective mass (*m*_e_*)		hole effective mass (*m*_h_*)	
crystal	Γ-A/Γ-Z	Γ-K/Γ-S	Γ-A/Γ-Z	Γ-K/Γ-S
NaLiZnS_2_	0.339	0.214	4.297	2.065
KLiZnS_2_ (indirect bandgap)	0.267	0.260	3.543	3.361
RbLiZnS_2_	0.217	0.216	0.867	0.885
CsLiZnS_2_	0.247	0.252	0.971	0.975

The band structure and density of states (DOS) near the Fermi level play crucial roles in enhancing both spintronic and thermoelectric performance. In thermoelectrics, a high DOS near the Fermi level often arising from flat bands or high valley degeneracy can significantly improve the Seebeck coefficient and power factor. This is because multiple degenerate valleys provide more states for charge carriers, increasing the DOS effective mass without severely affecting carrier mobility, thereby enhancing the overall thermoelectric efficiency (higher ZT). In spintronic materials, an ideal band structure features one spin channel (either up or down) crossing the Fermi level, while the other remains gapped. This results in high spin polarization at the Fermi level, which is essential for efficient spin injection, spin filtering, and achieving high tunnel magnetoresistance in devices. Thus, careful tuning of band structures and DOS near the Fermi level is key to optimizing the functional properties in both applications. Furthermore, phonon dispersion analysis reveals phonon bandgaps, which are associated with suppressed phonon scattering and reduced lattice thermal conductivity, an advantage for thermoelectric performance. Together, these electronic and vibrational features provide insights into the multifunctional potential of *M*LiZnS_2_ (M = K, Cs, and Rb).

PDOS in Figure 7a shows that in the trigonal structure of NaLiZnS_2_, *s* and *p* orbitals of both Na and Li contribute to the valence band around the Fermi level. The *p* and *d* orbitals of the Zn atom as well as the s orbitals of the S atom also contribute to the valence band around the Fermi level. The conduction band is dominated by the Zn-*d* and S-*p* orbitals. The *p* and *s* orbitals of Zn contribute significantly to the conduction band.

**Figure 7:**
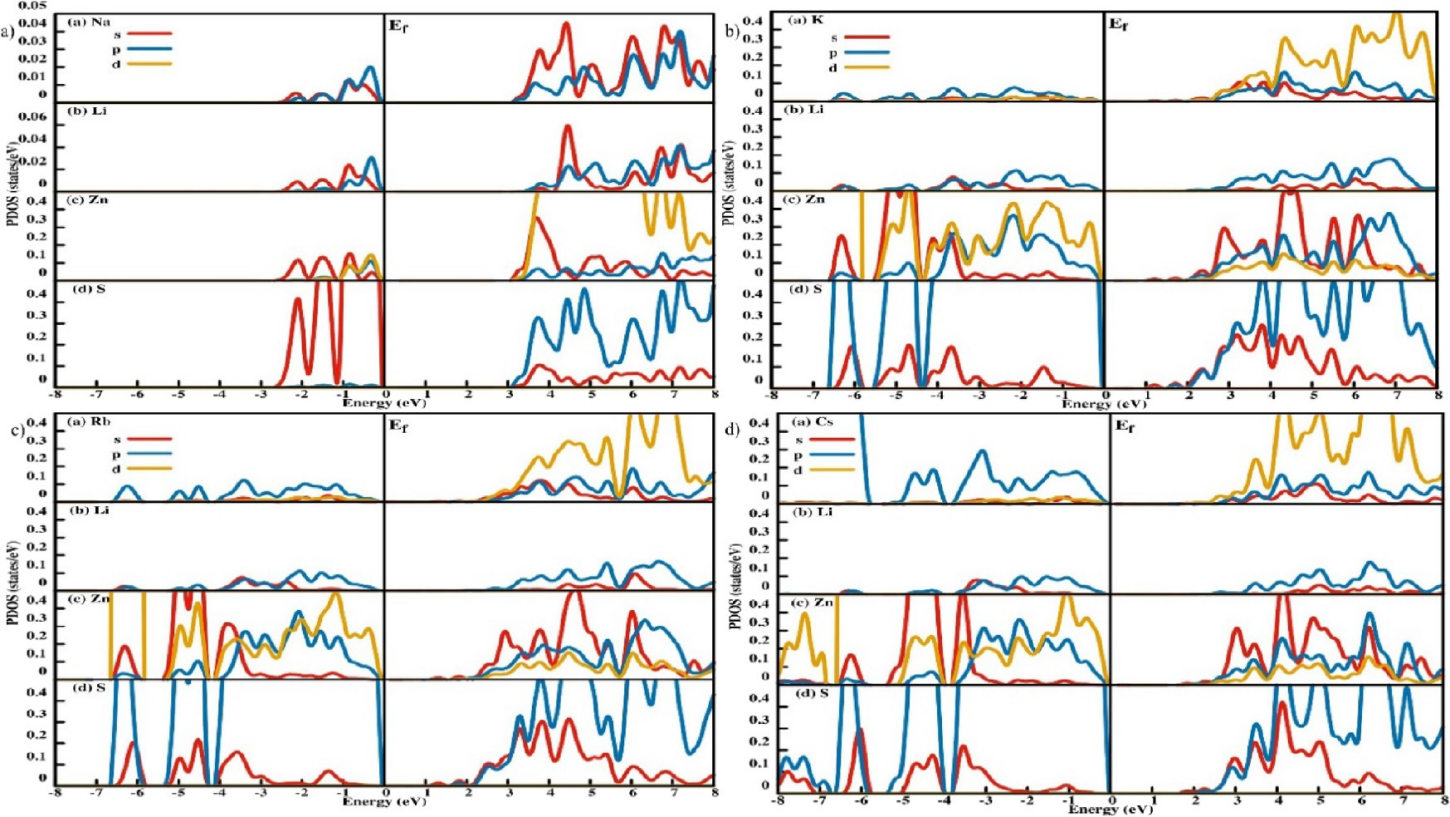
Partial Density of states (PDOS) for all eight structures of for *M*LiZnS_2_ series. (a) Top left image is for the trigonal structure of NaLiZnS_2_. (b) Top right-side image is for the tetragonal structure of KLiZnS_2_. (c) Bottom left side image is for the tetragonal structure of RbLiZnS_2_. (d) Bottom right image is for the tetragonal structure of CsLiZnS_2_.

Tetragonal *M*LiZnS_2_ (M = K, Rb, and Cs) PDOS is shown in Figure 7b. In these band structures, the *p* orbital of S atoms and the *d* and *p* orbitals of Zn atoms make up the majority of the Fermi level in the valence band. The *p* orbital contribution of the M and Li atoms in alkali metals is also significant in the valence band. The *d* and *p* orbitals of the M and S atoms contribute to the conduction band, and the *s* and *p* orbital states of the Zn atom have also been observed there. As the size of the *M* atom increases, the contribution of the *p* orbital of the *M* atom to the valence band at the Fermi level increases. The *s* and *p* orbital contributions of the Li atom to the valence band at the Fermi level are evenly distributed throughout all three configurations, independent of *M* atom size.

One could derive a bonding nature from the density of states. In NaLiZnS_2_, the *p* orbital of the S atom hybridizes with the *d* and *p* orbitals of the Zn atom, suggesting covalent bonding between Zn and S. At the same time, the relative number of S atom *p* states is significantly more than the *p* or *d* orbitals of the Zn atoms, indicating an ionic link between these two atoms. The bonding between Na–S and Li–S is also ionic in character since the contributions of Li and Na to S differ significantly. In tetragonal *M*LiZnS_2_ (M = K, Rb, and Cs), the bonding between Zn and S is primarily ionic, as indicated by the orbital contributions of the two atoms. However, as the M atom’s size increases, it tends to form stronger bonds with S atoms, which is evidenced by the increased *p*-orbital contribution of M at the Fermi level. Across all of these compounds, PDOS suggests that the Zn–S bonding strength is greater than that of M–S or Li–S.

The size of the M-site cation significantly influences the bonding characteristics in the four crystal structures examined. As the ionic radius grows, the bond lengths modified significantly, while the bond strength and nature change due to reduced orbital overlap and altered coordination geometry. The same kind effect is identified in perovskite magnetic materials recently that the size of the A-cation ionic radius modifies the geometry that eventually disturbs the balance between antiferromagnetic and ferromagnetic nature by modifying the strength of the exchange constant J and can even shift the magnetic ground state from antiferromagnetic to ferromagnetic, or to more complex spin arrangements.^34,35^ This change in lattice geometry also indirectly alters the electronic structure near the Fermi level, which in turn affects spin polarization and supports spin-dependent phenomena, such as Rashba or Dresselhaus band splitting. These effects arise not from the A-site cation directly, but rather from the distortion, it induces in the surrounding octahedral cage and accompanying symmetry breaking and spin–orbit interactions in halide perovskites.^36^

### 3.5 Vibrational Spectroscopy

One of the most efficient analytical methods employed to investigate the vibrational energy levels of molecules is vibrational spectroscopy. This approach focuses on studying the interaction between molecules and electromagnetic radiation, specifically with regards to their vibrational motions. When molecules absorb or emit electromagnetic radiation, they can switch between different vibrational states. This absorption or emission of light corresponds to specific energy changes within the molecule that can be detected and analyzed. Vibrational spectroscopy involves various techniques, including infrared spectroscopy (IR) and Raman spectroscopy, each of which provides unique information about molecular vibrations and the environment.

The normal modes of vibration of molecules and solids can be obtained from the group theory. For a system to be IR active, the dipole moment must change during vibrational excitation, and for Raman active (R), polarizability must change during vibrational excitation. Using group theory, IR and Raman active modes could be predicted from the symmetry of the system by knowing the point group symmetry of crystal structures from its space group. Irreducible representations in the particular character table that transform as *x*, *y*, and *z* are IR active and for Raman irreducible representations that transform like *xy*, *xz, yz, x* ^2^, *y* ^2^, *z* ^2^, and their linear combinations are active. In systems that have no centrosymmetric symmetry, both Raman and IR are active.

For NaLiZnS_2_, the point group symmetry is −3m (C^2^_3v_). This system has no centrosymmetric; thus, all IR modes are Raman active. From the character table, its IR and Raman active modes belong to A_1_ and E where A_1_ represents symmetric stretching and singly degenerate modes and E represents doubly degenerate vibrational modes. This crystal system has no Centro symmetry, and thus, all IR modes are also Raman active. The calculated active modes of IR and Raman are in Figure 8.

**Figure 8:**
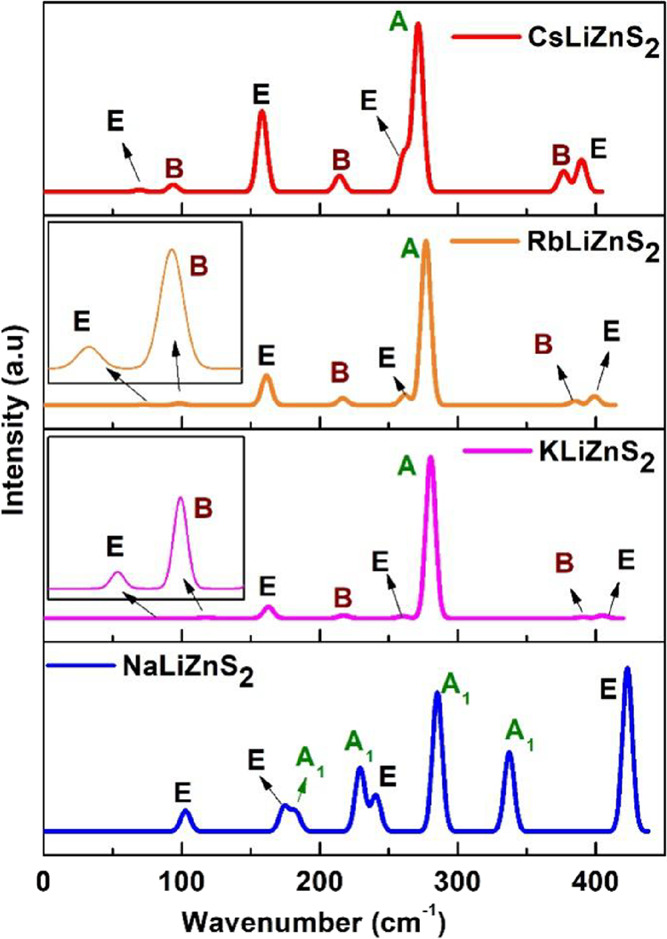
Calculated Raman spectrum for the equilibrium structure for series *M*LiZnS_2_.

For *M*LiZnS_2_ (M = K, Rb, and Cs), the point group symmetry is −4m2 (D^9^_2d_). This system has no Centro symmetry, and thus, all IR modes are also Raman active. There are three singly degenerate antisymmetric stretching (B) and four doubly degenerate stretching (E) that both are IR and Raman active. One symmetric stretching mode (A) is Raman active. The calculated and vibrational mode assigned for *M*LiZnS_2_ (M = K, Rb, and Cs) is shown in Figure 8. All peaks in KLiZnS_2_ structures move slightly toward a lower frequency in *M*LiZnS_2_ (M = Rb and Cs) when *M* atom is replaced by heavy alkali metal atoms. All peaks in KLiZnS_2_ become more dominant and visible in *M*LiZnS_2_ (M = Rb and Cs) due to the ionic radius size. Peaks around 404 and 390 cm^–1^ and 259 and 280 cm^–1^ in KLiZnS_2_ moves toward each other and get closer as the *M* atom’s size increases in *M*LiZnS_2_ (M = K, Rb, and Cs).

### 3.6 Optical Properties

DFT is a reliable and effective method for calculating the optical properties of quaternary semiconductors, which often show anisotropic behavior. Since measuring optical properties experimentally can be costly and time-consuming, DFT offers a quick and efficient way to predict these properties before material synthesis. The optical properties of semiconductors and metals are described by a frequency-dependent complex function called dielectric constant ε (ω), from which the linear response of materials is calculated when they interact with electromagnetic radiation. The dielectric function is written as ε­(ω) = ε_1_(ω) + iε_2_ω) in which real part ε_1_(ω) describes dispersion of electromagnetic radiation and imaginary part ε_2_(ω) describes radiation absorption by the material. These real and imaginary part can be derived from the Kramer–Kronig relationship.^37^

The linear optical constants such as reflectivity *R* (ω), refractive index *n* (ω), extinction coefficient *k* (ω), optical conductivity δ (ω), energy loss function *L* (ω), and absorption α (ω), which all can be derived from real part ε_1_(ω) and imaginary part ε_2_(ω), are related by the following equations:

$$R(\omega )={\left|\frac{\sqrt{\varepsilon_{1}(\omega )+i\varepsilon_{2}(\omega )}-1}{\sqrt{\varepsilon_{1}(\omega )+i\varepsilon_{2}(\omega )}+1}\right|}^{2}$$ 6

$$n\left(\omega \right)=\sqrt{\left(\frac{\sqrt{\varepsilon_{1}^{2}\left(\omega \right)+\varepsilon_{2}^{2}\left(\omega \right)}+\varepsilon_{1}\left(\omega \right)}{2}\right)}$$ 7

$$k\left(\omega \right)=\sqrt{\left(\frac{\sqrt{\varepsilon_{1}^{2}\left(\omega \right)+\varepsilon_{2}^{2}\left(\omega \right)}-\varepsilon_{1}\left(\omega \right)}{2}\right)}$$ 8

$$\delta (\omega )=\frac{\omega }{4\pi }\varepsilon_{2}(\omega )$$ 9

$$L\left(\omega \right)=\frac{\varepsilon_{2}\left(\omega \right)}{\varepsilon_{1}\left(\omega \right)+\varepsilon_{2}\left(\omega \right)}$$ 10

$$a\left(\omega \right)=\frac{\omega }{c}\sqrt{2\left(\sqrt{\varepsilon_{1}^{2}\left(\omega \right)+\varepsilon_{2}^{2}\left(\omega \right)}-\varepsilon_{1}\left(\omega \right)\right)}$$ 11

The optical properties of semiconductors are determined by their ability to absorb and emitting of light. Semiconductors have a limited range of absorption and emission spectra due to the energy bandgap between the valence and conduction bands. Semiconductors are important for a variety of applications including solar cells, LEDs, and lasers.

The calculated optical properties of the new series *M*LiZnS_2_ are presented in Figure 9 and in the Supporting Information (S1–S3). The dielectric function of *M*LiZnS_2_ (M = Na, K, Rb, and Cs) was calculated across a photon energy range of 0–40 eV. For NaLiZnS_2_, ε_1_(ω) showed two distinct peaks at 6 and 7 eV, corresponding to interband transitions. In the K, Rb, and Cs variants, a broad peak was observed at ∼6 eV, and the high-energy peak around 20 eV split into two distinct peaks as the cation size increased. The imaginary part ε_2_(ω) was zero below the bandgap, confirming the semiconducting nature, and showed absorption peaks corresponding to the transitions in ε_2_(ω). These variations highlight the influence of the ionic size of M on the optical properties of the *M*LiZnS_2_ series. The dual peaks in NaLiZnS_2_ can arise from its tighter bonding and more distinct electronic states due to the smaller ionic radius of Na, whereas the broader single peak in K, Rb, and Cs variants reflects the impact of larger cations on lattice parameters, bonding, and electronic transitions. This emphasizes the sensitivity of the optical properties to minor structural and compositional changes. The difference in the optical properties of the NaLiZnS_2_ variant compared to the other members of the *M*LiZnS_2_ series (M = K, Rb, and Cs) can be directly linked to its distinct electronic structure, as revealed by our calculations. This behavior reveals the ability to modify optical properties via ionic substitution, making these materials highly adaptable for use in optoelectronic devices.

**Figure 9:**
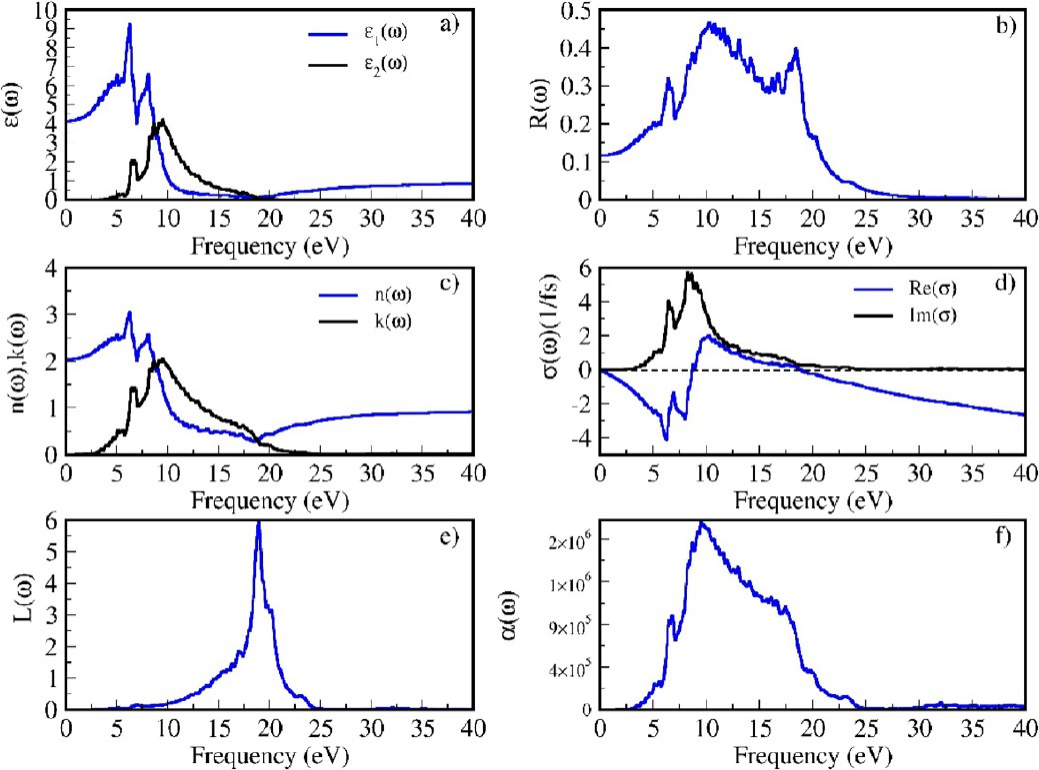
Calculated optical properties for NaLiZnS_2_. In each image of the series calculated optical properties for the *M*LiZnS_2_ series (given in S1, S2, and S3 of the Supporting Information): (a) dielectric function ε (ω), (b) reflectivity *R* (ω), (c) refractive index *n* (ω); extinction coefficient *k* (ω), (d) optical conductivity δ (ω), (e) energy loss function *L* (ω), and (f) absorption α (ω).

From the dielectric constant, the refractive index of the series that can be calculated at an infinite wavelength of NaLiZnS_2_ is about 2.1 eV (Figure 8) and for other materials in this series is about 1.8 eV (S1, S2, S3 of the Supporting Information). A lower refractive index in KLiZnS_2_, RbLiZnS_2_, and CsLiZnS_2_ indicates reduced reflection and potentially enhanced transparency at longer wavelengths compared to NaLiZnS_2_. This could make these materials more suitable for applications requiring high transmission of low-energy radiation. The extinction coefficient represents how effectively the material can absorb and dissipate the energy of interacting electromagnetic waves. Since k­(ω) is closely related to ε_2_(ω), the peaks observed in k­(ω) correspond to features in ε_2_(ω), which are associated with electronic transitions between valence and conduction bands or intraband transitions. The extinction coefficient exhibits a broad peak in the range of 3–25 eV in NaLiZnS_2_. In KLiZnS_2_, the extinction coefficient exhibits a broad peak in the energy range of approximately 3–15 eV and a smaller peak in the range of 17–25 eV. The smaller peak observed in KLiZnS_2_ between 17 and 25 eV becomes more prominent and shifts toward 15 eV in RbLiZnS_2_ and CsLiZnS_2_. This indicates a change in the optical absorption behavior, most likely caused by changes in the electronic structure of the material as the alkali metal atoms (M) transition from K to Rb and Cs.

In the reflectivity spectrum, a broad reflectivity peak is observed spanning the energy range of 0–25 eV NaLiZnS_2_. For KLiZnS_2_, two distinct peaks are observed, one main peak at about 12 eV and another small peak at about 22 eV. The presence of multiple peaks indicates more selective reflectance behaviors at these specific energy levels, potentially due to electronic transitions or structural influences introduced by potassium. This small peak becomes sharper and becomes the dominant one in *M*LiZnS_2_ (M = Rb and Cs). The reflectivities at an infinite wavelength for the series *M*LiZnS_2_ (M = Na, K, Rb, and Cs) are 12, 8, 9, and 9%, respectively. In photovoltaic solar cells (PVS), the concept of antireflection coatings (ARC) is widely utilized, particularly in silicon-based PVS, to enhance their efficiency. Silicon-based PVS can harness only about 60% of the solar spectrum, while the remaining 40% is lost primarily due to reflectance. To minimize these losses, thin layers of wide-bandgap semiconductors, such as TiO_2_ and SiO_2_, are coated on the silicon surface. These coatings have been shown to significantly improve the overall performance of the cells. The key requirements for an effective antireflection coating are a low absorption coefficient throughout the visible and UV regions and an appropriate refractive index to optimize light transmission and minimize reflection. Considering these criteria, the present series (*M*LiZnS_2_) exhibits a wide bandgap and a suitable refractive index, making it a promising candidate for antireflection coating applications in photovoltaic solar cells. The efficiency of PV solar cells is significantly influenced by the properties of the ARC materials.

The following equation shows the relationship between optimal wavelength and thickness:

λ=4nd 12

where λ is optimal wavelength for silicon solar cell 500 nm, *n* is refractive index of ARC material, and *d* is the thickness of ARC. For the present series (*M*LiZnS_2_), the calculated optimal thickness of ARC to transfer most of the solar spectrum in visible and UV region is 694 nm. *M*LiZnS_2_ could be a superior choice for its broader spectral absorption.

Optical conductivity plot δ (ω) shows that the real part has only one peak in the range of 0–10 eV, which reaches a negative value of about 9 eV for almost all materials in *M*LiZnS_2_. The energy loss of fast-moving electrons in a material is described by the electron energy loss function *L* (ω). NaLiZnS_2_ has one sharp peak at 17 eV and in KLiZnS_2_, another peak appears at 23 eV, and this peak becomes more dominant than the 17 eV peak in RbLiZnS_2_ and CsLiZnS_2_.

All four materials exhibit strong absorption capabilities in the low-frequency range of 3–10 eV. NaLiZnS_2_ displays a broad absorption peak between 3 and 20 eV, while this peak splits into two sharper peaks in KLiZnS_2_. In the case of *M*LiZnS_2_ (where *M* = Rb and Cs), the second peak becomes the dominant feature. The absorption edges for the series are observed at 2.91, 3.58, 3.55, and 3.61 eV for each material, aligning well with their calculated bandgaps. This behavior occurs because the electrons in the valence band begin interacting with the incoming radiation when the fundamental gap matches the energy of the incident electromagnetic radiation. For all four materials in this new series, absorption reaches its peak at 10 eV and decreases with further increases in the energy of the incident radiation. Two distinct peaks are observed: one at 10 eV and another at 17.5 eV. The second peak at 17.5 eV becomes more prominent as the size of M increases. These characteristics suggest that the series may be suitable for optical applications within this energy range. Additionally, the low reflectivity and relatively low absorption in the 3–5 eV range indicate that these materials could be used as ARC materials, similar to TiO_2_ and SiO_2_, in photovoltaic solar cells, particularly for UV–vis applications.

### 3.7 Transition Dipole Moment

The transition dipole moment is a fundamental quantity governing light-matter interactions, particularly within the context of electronic band structures. It directly determines the probability of electronic transitions, enabling the calculation of the absorption and emission spectra. These spectra provide crucial insights into a material’s electronic structure and consequent properties, such as conductivity and optical behavior. Furthermore, the transition dipole moment plays a critical role in understanding light-induced processes, such as photodissociation and photoexcitation. The transition dipole moments are calculated between the occupied and unoccupied Kohn–Sham orbitals. This includes evaluating the integral of the dipole moment operator between the relevant electronic states. Knowledge of transition dipole moments helps in the development of efficient solar cells, LEDs, and lasers by selecting materials with favorable optical properties.

The optical density of states for *M*LiZnS_2_ (M = Na, K, Rb, and Cs) is shown in Figure 10 a–d. The available states between the valence and conduction bands, which are crucial for optical absorption and emission processes in semiconductors, can be qualitatively understood from the joint density of states (JDOS). JDOS describes the interband transitions between the valence and conduction bands, which determine the suitability of a semiconductor for optical applications. In NaLiZnS_2_, small cascade peaks are observed between 0 and 10 eV, indicating a greater number of available states for transitions during optical absorption and emission. Among the other three materials, KLiZnS_2_ exhibits relatively more states for optical transitions. When considering application-related issues, intraband transitions that limit the optical transition process should ideally have fewer available states. The partial joint density of states (PJDOS) for the *M*LiZnS_2_ series (M = Na, K, Rb, and Cs) is shown in Figure 11a–d. While all four materials exhibit fewer available states for intraband transitions, CsLiZnS_2_ has the fewest, with only three peaks. In this series, intraband transitions have fewer available states compared to interband transitions. Specifically, CsLiZnS_2_ displays a larger number of interband transition states. This indicates that intraband transitions are significantly smaller than interband transitions in this series, suggesting its potential suitability for applications related to solar cells.

**Figure 10:**
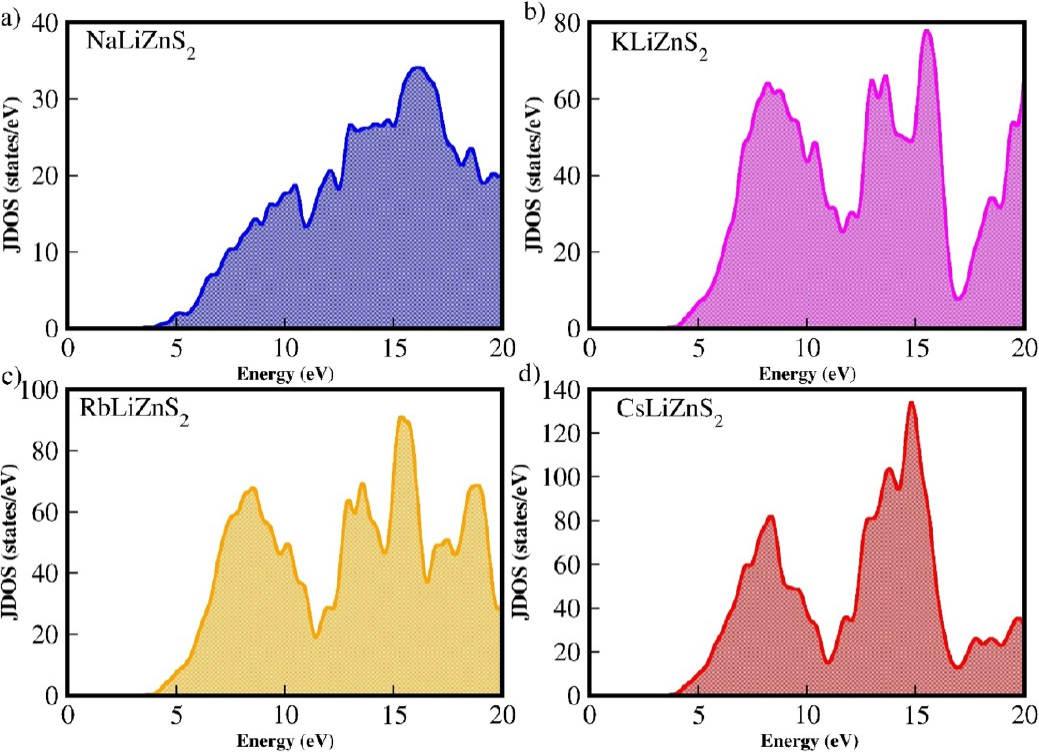
Calculated JDOS for the *M*LiZnS_2_ (*M* = Na, K, Rb, and Cs) series: (a) NaLiZnS_2_, (b) KLiZnS_2_, (c) RbLiZnS_2_, and (d) CsLiZnS_2_.

**Figure 11:**
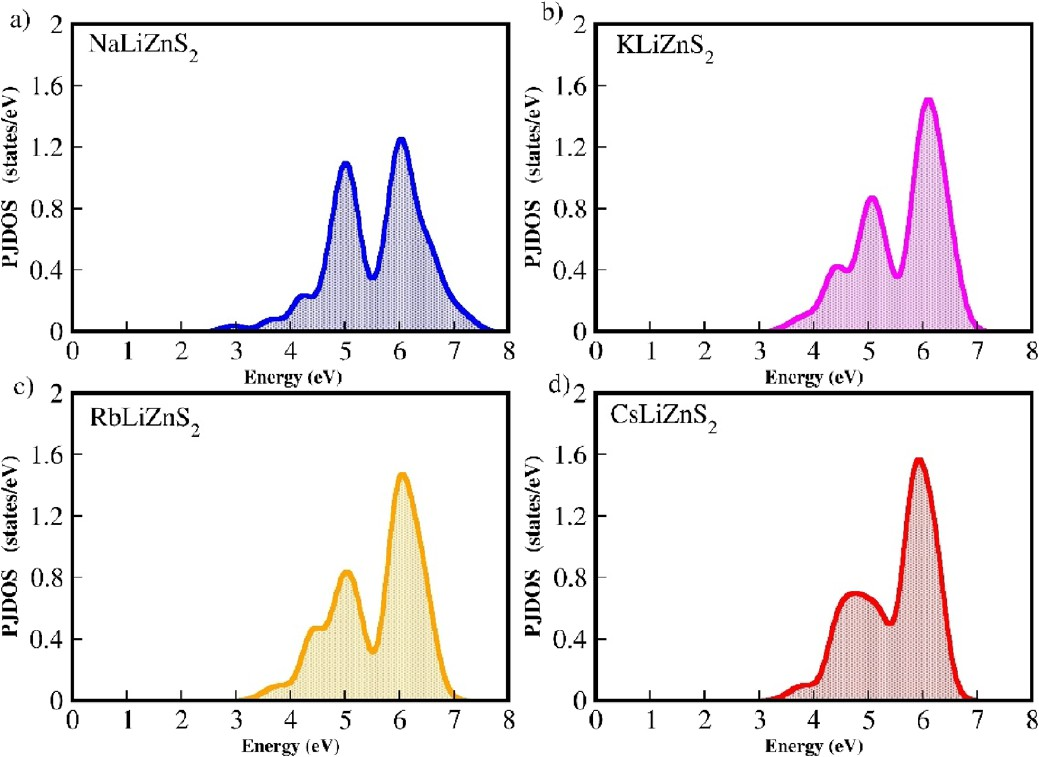
Calculated PJDOS for of the *M*LiZnS_2_ (M = Na, K, Rb, and Cs) series. (a) NaLiZnS_2_, (b) KLiZnS_2_, (c) RbLiZnS_2_, and (d) CsLiZnS_2_.

The transition dipole moment is calculated and illustrated in Figure 12a–d. The probability of electron transitions from valence band states to conduction band states is determined by the dipole transition states. The square of the transition dipole moment between a valence band and a conduction band quantifies the strength of optical transitions, as it reflects the charge distribution’s interaction with electromagnetic radiation.

**Figure 12:**
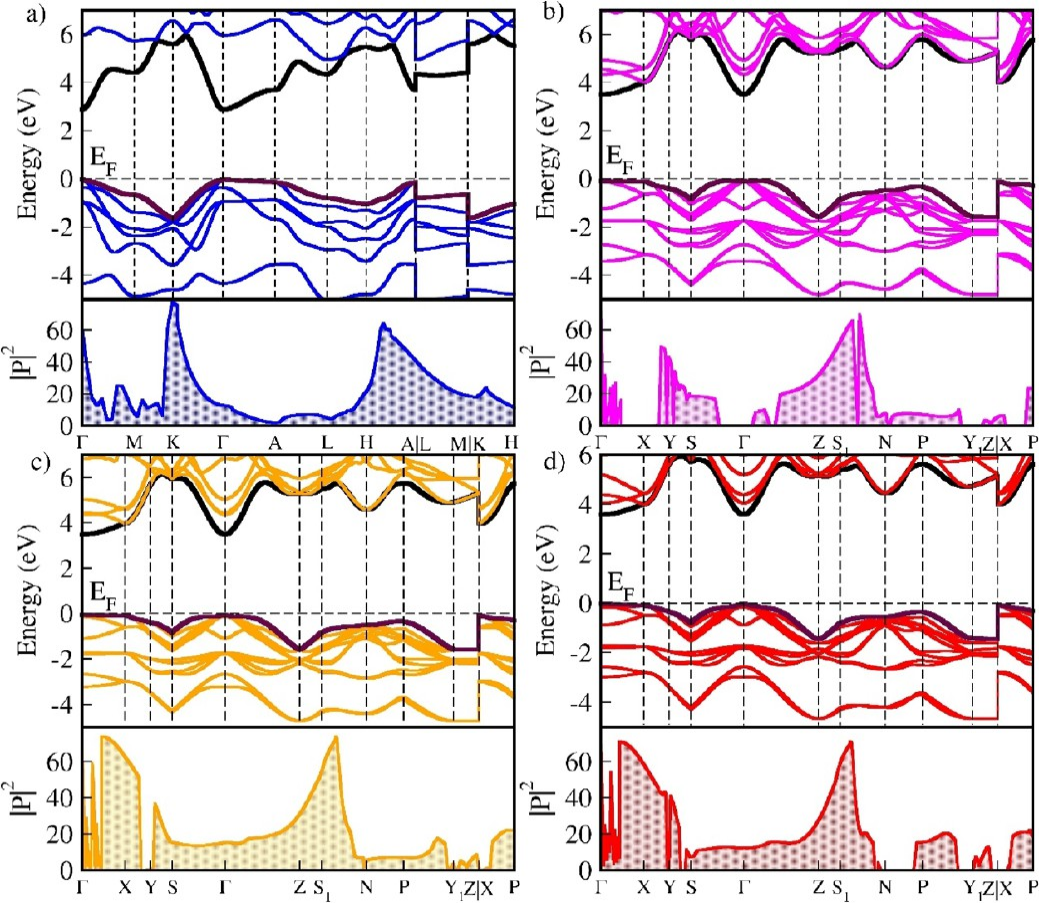
Calculated band structures and transition matrix elements for *M*LiZnS_2_ (M = Na, K, Rb, and Cs) series: (a) NaLiZnS_2_, (b) KLiZnS_2_, (c) RbLiZnS_2_, and (d) CsLiZnS_2_. Black line represents a conduction band, and maroon color represents a valence band in the picture.

In the case of NaLiZnS_2_, the transition dipole moment along the Γ–A high-symmetry path decreases to zero, indicating that the valence band flattens in this direction, leading to a low transition probability. In KLiZnS_2_, the transition dipole moment suggests an indirect transition, as the probability of transition at Γ is zero, while a strong transition is observed along the Z–S direction. For the other two structures RbLiZnS_2_ and CsLiZnS_2_, the transition probability suggests that they could exhibit direct bandgap behavior, as they show significant transitions near the Γ–Z direction. Additionally, the integration of |P|^2^ curves reveals that CsLiZnS_2_ has a higher transition probability than the other structures. The cumulative sum of the integrated |P|^2^ values for *M*LiZnS_2_ (*M =* Na, K, Rb, and Cs) is 106.36, 81.31, 108.37, and 111.45, respectively.

### 3.8 Effective Mass

The effective mass of the electron and hole of this new series is calculated using vaspkit^38^ for valence band maximum to conduction band minimum and tabulated in Table 4. The effective mass of charge carriers plays a crucial role in determining the transport and optical properties of the materials. The hole effective mass (*m*_h_^*^) represents the effective mass of a “hole”, which is defined as the absence of an electron in a material. Similarly, the electron effective mass (*m*_e_^*^) characterizes the effective mass of an electron within the system. The mobility of charge carriers in semiconductor materials is governed by the curvature of the electronic band structure, which is quantified by the effective mass. The effective mass is determined by the curvature of the valence band maximum and the conduction band minimum. A larger curvature at these points corresponds to a lower effective mass, resulting in a higher carrier mobility. In the case of NaLiZnS_2_, the effective mass along the Γ–A direction is significantly higher compared to the Γ–K direction. This is attributed to the band structure characteristics, where both the valence and conduction bands exhibit a relatively flat dispersion along the Γ–A. A flatter band corresponds to a lower band curvature, which results in a higher effective mass, thereby reducing the carrier mobility in this direction. Conversely, in the Γ–K direction, the bands exhibit a greater curvature, leading to a lower effective mass and, consequently, higher mobility of the charge carriers. For the other three structures—KLiZnS_2_, RbLiZnS_2_, and CsLiZnS_2_—the valence band shows a higher degree of curvature, leading to a lower hole effective mass and, thus, improved hole mobility. However, among these three, KLiZnS_2_ exhibits the highest hole effective mass due to its indirect bandgap nature, where the valence band has a lower curvature. This implies that hole mobility in KLiZnS_2_ is significantly reduced compared to those in the other structures. In contrast, RbLiZnS_2_ and CsLiZnS_2_ exhibit both high electron and hole mobilities, suggesting superior charge transport characteristics in these materials. The effective mass of electrons is usually smaller than that of holes, resulting in higher mobility and faster response for electrons. This difference significantly affects material properties, with smaller hole effective masses leading to better electrical conductivity and smaller electron effective masses enhancing thermoelectric performance. The increased band curvature in these structures leads to lower effective masses for both electrons and holes, enhancing their transport properties. These features indicate that RbLiZnS_2_ and CsLiZnS_2_ could serve as promising candidates for applications requiring efficient charge carrier dynamics, such as optoelectronic and thermoelectric devices.

**Table 5:** Calculated Bandgaps E_g_ (eV), Chemical Hardness $\eta =\frac{E_{g}}{2}\left(\mathrm{eV}\right)$ (eV), and Global Softness *S* = 1η (eV^–1^) for the *M*LiZnS2 (M = Na, K, Rb, and Cs) Series

compound	E_g_ (eV)	$\eta =\frac{E_{g}}{2}\left(\mathrm{eV}\right)$	$S=\frac{1}{\eta }({\mathrm{eV}}^{-1})$
NaLiZnS_2_	2.913	1.456	0.686
KLiZnS_2_	3.581	1.790	0.558
RbLiZnS_2_	3.552	1.7763	0.5629
CsLiZnS_2_	3.606	1.803	0.554

To gain further insights into the chemical reactivity and stability of the studied compounds, we evaluated selected conceptual CDFT descriptors derived from the electronic structure. Since the vacuum alignment (work function, Φ) is not available in the present study, we restrict our analysis to the vacuum-independent quantities: the bandgap (E_g_), chemical hardness $\eta =\frac{E_{g}}{2}$ and global softness $S=\frac{1}{\eta }$ . These descriptors provide a direct measure of resistance and susceptibility to charge transfer and can be obtained solely from the calculated bandgaps. Global reactivity parameters such as electronegativity (χ), chemical hardness (η), softness (*S*), and electrophilicity index (ω) provide a conceptual framework linking electronic structure to photovoltaic performance.^39^ Table 5 shows the calculated vacuum-independent quantities for the *M*LiZnS_2_ (M = Na, K, Rb, and Cs) series. For example, the decrease in hardness from NaLiZnS_2_ to CsLiZnS_2_ is consistent with enhanced polarizability and a stronger optical response in heavier members. The electrophilicity index underscores their electron-accepting character, relevant for electron-transport and antireflective coating functions in solar cells. Values of electronegativity (χ) and electrophilicity (ω) are not reported since the work function (Φ) is unavailable. For completeness, we also provide the relations χ = Φ – Δ_mid_ and $\omega =\frac{\chi^{2}}{2\eta }$ , where Δ_mid_ = (*E*_CBM_ – *E*_VBM_)/2 – *E*_*F*_, which may be applied once Φ becomes available. For RbLiZnS_2_, using *E*_VBM_ = 0.4973 eV (*E*_CBM_ = 4.0500 eV and *E*_F_ = 0.5723 eV), we obtain E_g_ = 3.5527 eV, η = 1.77635 eV, *S* = 0.5629 eV^–1^, and Δ_mid_ = 1.70135 eV.

## 4 Conclusions

Our first-principles study establishes that *M*LiZnS_2_ (M = Na, K, Rb, and Cs) undergoes a structural transition from trigonal (NaLiZnS_2_) to tetragonal (K, Rb, and Cs) phases, governed by cation size. Phonon dispersion and elastic constants confirm their dynamic and mechanical stabilities (except tetragonal NaLiZnS_2_). Hybrid functional bandgaps (2.9–3.6 eV) match experimental trends in related systems, supporting their semiconducting character. The optical analysis demonstrates low reflectivity and refractive indices suitable for antireflection coatings in photovoltaic devices. Flat valence bands and phonon gaps point to high hole mobility and reduced thermal conductivity, linking these compounds to spintronic and thermoelectric potential. This combined structural-electronic-optical framework offers a computational foundation to guide future experimental studies on *M*LiZnS_2_ and related layered chalcogenides for multifunctional energy applications.

## Supplementary Material



## Notes

Calculated optical properties of *M*LiZnS_2_ (M = K, Rb, and Cs), including dielectric function ε­(ω), reflectivity R­(ω), refractive index n­(ω), extinction coefficient k­(ω), optical conductivity σ­(ω), energy loss function L­(ω), and absorption coefficient α­(ω) (Figures S1–S3) (PDF)
