# Supplementary material for: A New Series of Multication MLiZnS2 (M = Na, K, Rb, and Cs) Compounds for Photovoltaic Applications—A First-Principles Study
Source: ACS Omega. 2025 Oct 14;10(42):49924–40. doi: 10.1021/acsomega.5c06023 (PMC12572982; doi:10.1021/acsomega.5c06023)
Supplement: Supplementary file 1 [file ao5c06023_si_001.pdf]

# Supporting Information

# A new series of multi-cation *M*LiZnS$_2$ (*M* = Na, K, Rb, Cs) compounds for photovoltaic applications – a first-principles study

Suresh Alagarsamy [1] Kanimozhi Balakrishnan[2] Ponniah Vajeeston[*3]

[1]*Department of Physics, Maulana Azad National Institute of Technology, Bhopal, Madhya Pradesh,462003, India.*

[2]*Department of Computational Physics, School of Physics, Madurai Kamaraj University, Madurai 625021, Palkalai Nagar, Tamil Nadu, India.*

[3]*Department of Chemistry and Center for Materials Science and Nanotechnology, University of Oslo, Oslo 0371, Norway.*

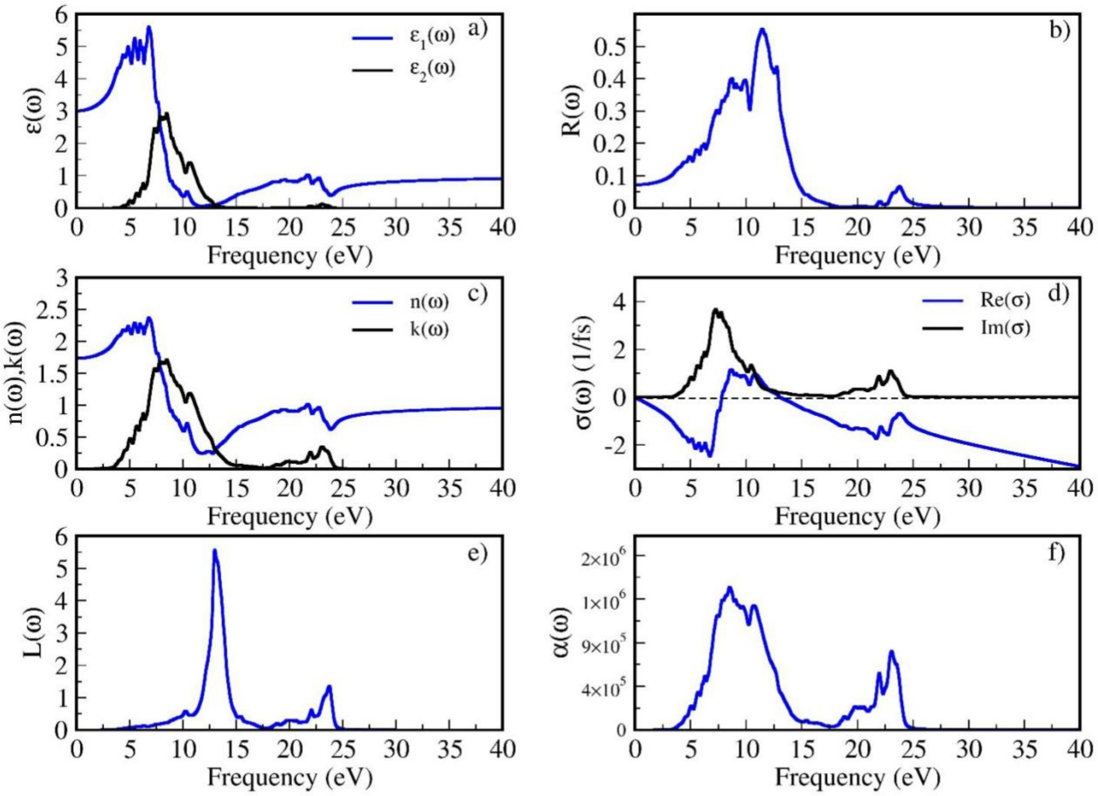


FigureS1: Calculated optical properties for KLiZnS$_2$. In each image of the series calculated optical properties for *M*LiZnS$_2$ series (a) dielectric function ε(□), (b) reflectivity R(□), (c) refractive index n(□); extinction coefficient k(□), (d) optical conductivity δ(□), (e) energy loss function L(□), and (f) absorption α(□).

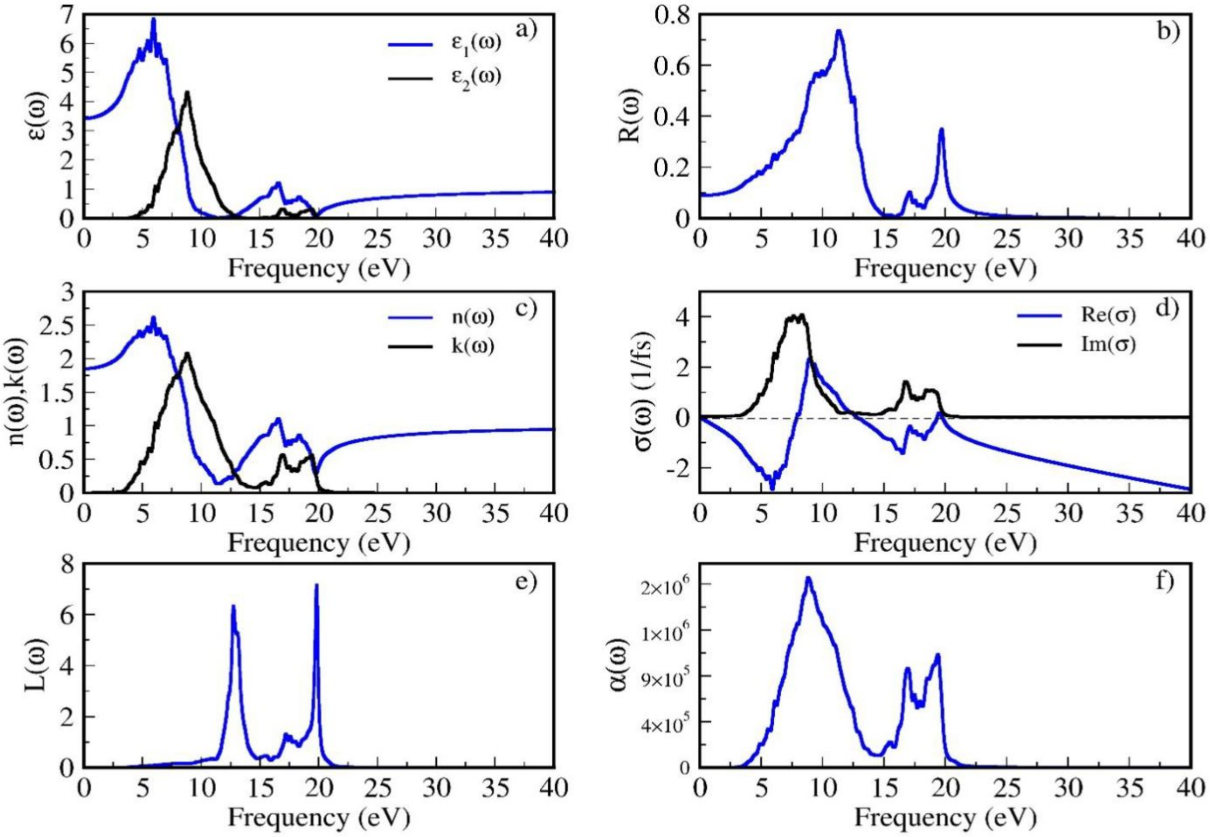


FigureS2: Calculated optical properties is for $RbLiZnS_2$. In each image of the series calculated optical properties for $M$$LiZnS_2$ series (a) dielectric function ε(□), (b) reflectivity R(□), (c) refractive index n(□); extinction coefficient k(□), (d) optical conductivity δ(□), (e) energy loss function L(□), and (f) absorption α(□).

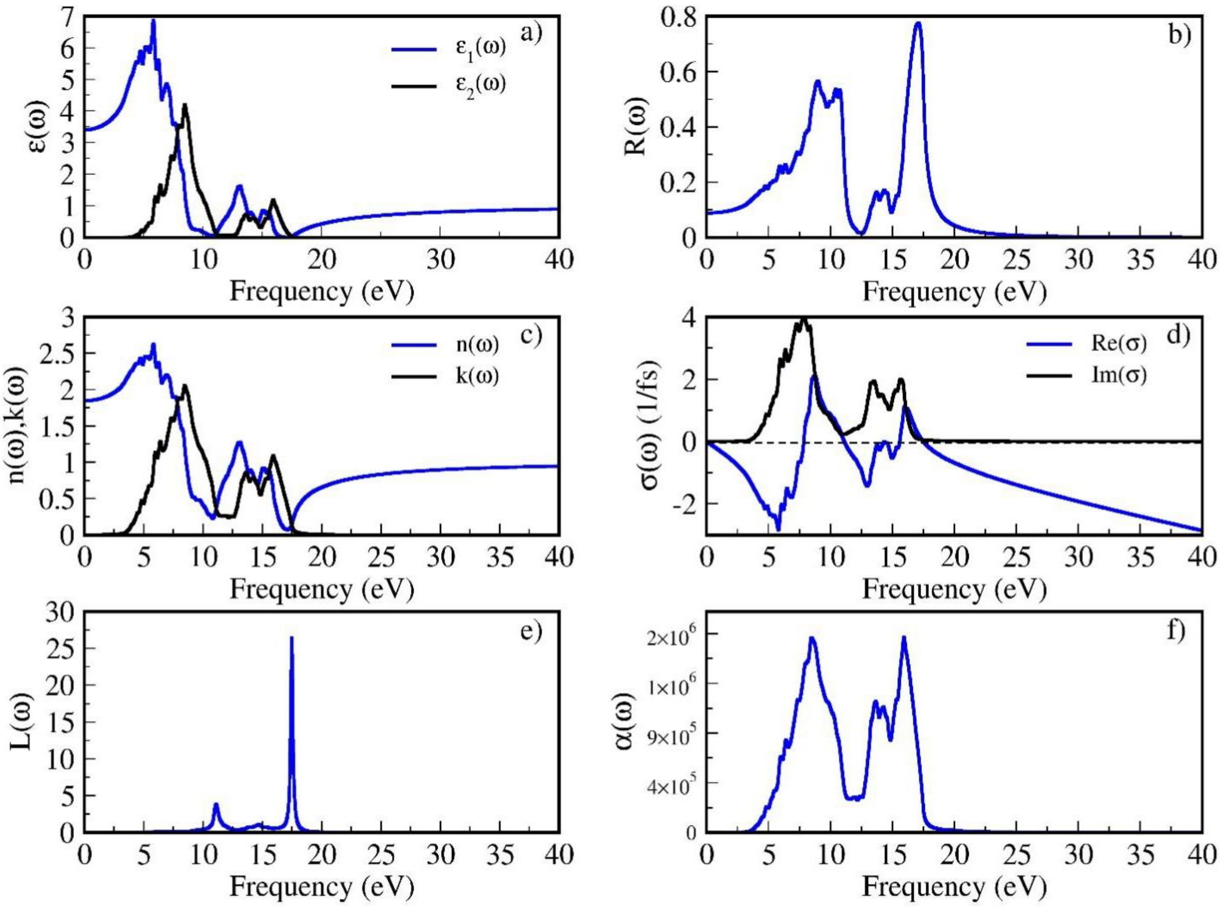


FigureS3: Calculated optical properties for $CsLiZnS_2$. In each image of the series calculated optical properties for $M$Li$ZnS_2$ series (a) dielectric function ε(□), (b) reflectivity R(□), (c) refractive index n(□); extinction coefficient k(□), (d) optical conductivity δ(□), (e) energy loss function L(□), and (f) absorption α(□).
